# Impacts of educational interventions of librarian instruction on health information seeking attitudes and behaviors in an employee wellness program

**DOI:** 10.5195/jmla.2024.1775

**Published:** 2024-04-01

**Authors:** Colleen Marie Foy

**Affiliations:** 1 foyc@wfu.edu, Research and Instruction Librarian for the Sciences, Zachary Smith Reynolds Library, Wake Forest University, Winston-Salem, NC

**Keywords:** Health literacy, instruction techniques, employee wellness, health promotion, library partnership, academic library

## Abstract

**Objective:**

Health literacy and its potential impacts on the wellbeing of patrons remain a highly regarded objective among health science and medical librarians when considering learning outcomes of patron communities. Librarians are positioned to champion literacy instruction activities. This study aimed to examine health information seeking attitudes and behaviors in an academic-based employee wellness program before and after health literacy workshops were developed and facilitated by an academic health sciences librarian.

**Methods:**

The intervention included instruction informed by Don Nutbeam's Health Literacy Framework and the Research Triangle Institute's Health Literacy Conceptual Framework. Sixty-five participants obtained through convenience sampling attended workshops and were invited to respond to pre- and post-session surveys. Using a quantitative quasi-experimental methodology, surveys collected health literacy indicators including preferred sources and handling practices of in-person and online health information.

**Results:**

Findings indicated workshops influenced information seeking behaviors as participants documented a decrease in social media use for health and wellness information (-36%) and medical information (-13%). An increase in the usage of consumer health databases (like Medline Plus) was also indicated post-workshop for health and wellness information (18%) and medical information (31%).

**Conclusion:**

Favorable impacts are evident in this small-scale study; however, more research is needed to confirm the influence of these methods on larger and more diverse populations. Librarians should continue to develop and disseminate theory-informed tools and methods aimed at engaging various communities in constructive health information seeking practices.

## INTRODUCTION

Information literacy remains the core objective for librarians, particularly for instructional librarians working in academic settings. Learning outcomes related to literacy and its associated behaviors and attitudes are a common thread throughout library instructional sessions. Health literacy (HL) and its potential impacts on patron wellbeing also remain a specific objective for health science librarians when working with student and patient communities. The Centers for Disease Control and Prevention has found that nearly 90% of the United States adult population has limited HL [1] and the United Nations recently declared the COVID pandemic to be an infodemic [2]. The global wellness market exceeds $5 trillion, of which $1.9 billion is spent in the US on traditional medicine, weight loss, nutrition, physical activity, and preventative medicine products and services [3], and mobile phone research suggests users receive an average of 74 notifications per day [4]. This context has created a strong need for valid health information [5] and increased literacy to help students, patients, and patrons navigate through this information landscape.

The phrase “health literacy” dates back to the 1970s when it used to describe standards for grade level education [6] and in the publication “Healthy People: The Surgeon General's Report on Health Promotion and Disease Prevention” which elicited the Healthy People Initiatives [7] in the United States, its definition has evolved to now include both personal and organizational responsibilities in the pursuit of public health [8]. With US adults spending over half their waking hours in the workplace [9] and the World Health Organization recognizing employee wellness programs as a best buy platform for non-communicable disease prevention and control [10], corporate wellness programs have great potential for the proliferation of health literacy concept awareness and skill development. Workplace wellness programs can include health risk assessments, educational sessions, exercise and weight loss motivated activities, nutrition counseling, health fairs, and disease targeting [11–16]. The promising synergies of combining the reach of existing workplace wellness programming with a health science librarian's unique skills as health information professional, subject specialist, fact checker, and facilitator of health literacy skill development [17–19] are worth exploring.

This study's intervention is based upon Don Nutbeam's 2000 Health Literacy Model and the Research Triangle Institutes (RTI) Health Literacy Conceptual Framework of 2012. The Health Literacy Model explores HL as a function of wellbeing and everyday life and establishes a hierarchical lens on related concepts. Each level - functional, interactive, and critical – promotes independence and empowerment of health information consumers [5]. The RTI's Health Literacy Conceptual Framework bridges the gap between form and function of HL and acknowledges the complexity and range of individual HL skill attainment. It theorizes that demographic characteristics, prior knowledge and experiences, accessibility of resources, physical and intellectual capabilities, as well as external factors such as mass media, which now includes the internet and social media, influence the development, refinement, and utilization of health literacy skills to varying degrees [20].

Numerous examples of library educational partnerships are evident in the literature on HL education programming and support in public, academic, and special library settings. While playing “neutral party” and “connector” roles as multidisciplinary partners and information disseminators, librarians are model players when engaging in and developing partnerships both internally and externally to advance literacy among patron groups [21]. Barr-Walker et al. [22], Shipman et al. [21], and Swanberg et al. [23] published overviews of specific and successful instances of library involvement in HL support programs. From developing workshops with objectives to decrease computer anxiety in older adults and refugee populations when making health decisions, to workshops and tool kits designed for local community groups in support of valid health information seeking behaviors, to building tutorials that expand HL and evidence-based medicine concepts for health science students, librarians are empowering patients as consumers and students as future educators and providers. To highlight collaborative examples targeting young adults, but with highly adaptable and transferable curriculums and objectives for diverse populations, health science and medical libraries in Texas [24] and Maryland [25] developed or supported HL modules to strengthen those skills in high school students. Both programs met students in their settings and facilitated interactive real-life examples which trained students to locate and evaluate online health information and used gaming to reinforce concepts. Both programs also increased confidence levels for a diverse population of young adults to use and validate online health information [24,25].

Although the benefits of workplace wellness programming are highly debated in the literature relative to returns on investment, workforce health risks and productivity, and employee moral [11,13,15,26], specific instances of programs that apply HL-focused constructs may yield isolated, but measurable, outcomes. Literacy-focused interventions have been shown to influence diet choice and quality outcomes in workforce populations following educational workshops and messaging strategically scheduled and placed in workplace environments [12,14]. To address overall HL aptitude among employee organizations, a 2016 Virginia Tech study determined that existing HL levels among employees from various backgrounds and professional settings may influence the likelihood of participation and success at each phase of programming. Although employees with limited HL were less likely to enroll in programs offered and achieve prescribed weight loss objectives [16]. In each study case, results convey the need for strategies that enhance HL levels in workplace settings to improve participation in health-driven incentive programs and their potential participation-related health outcomes.

In a 2020 bibliometric analysis, Wilson et al. posited that the lack of published HL related work by librarians may not directly correlate to the successful execution of those efforts within their communities[27]. It is hoped that increased publication of work in this field may facilitate the dissemination of successful approaches and applications of HL instruction. Therefore, with improved HL linked with favorable health outcomes, and the lack of HL concepts and instructional strategies evident in the employee wellness literature, this study aimed to answer the following research questions:

- What is the impact of theory informed librarian-facilitated instruction on understanding and attitudes toward HL in an employee wellness program through intentional instructional design and behavioral assessment?
- What is the impact of experiential learning approaches on health information evaluation behaviors?
- What is the impact of exposure to valid health information sources on the likelihood of user engagement?

## METHODS

### Design and Sample

Taking a quantitative approach to a quasi-experimental methodology (without a control group), instruction and pre-post surveys were designed for participants in predetermined cohorts of a medically directed employee wellness program at Wake Forest University (WFU). The Health and Exercise Science (HES) Department at WFU conducts Healthy Exercise & Lifestyle ProgramS (HELPS) which offer a three-month Therapeutic Lifestyle Change (TLC) program and a six-month Healthy Weight for You (H-WFU) program. HELPS session schedules, participants, formats, and durations were predetermined by administrators prior to library involvement; therefore, the librarian investigator did not dictate nor participate in sampling practices. All full-time WFU faculty and staff employees may participate in HELPS programming as space allows. Although the WFU Office of Institutional Research publishes employment demographic statistics [28], that information was not collected during this research and may or may not be representative of overall workshop participants. Sixty-five individuals attended HL workshops during the Fall 2022 and Spring 2023 semesters: Fall TLC (n=15), Fall H-WFU (n=17), Spring TLC (n=15), Spring H-WFU (n=18). Although the study investigator acts as the HES librarian liaison, project advocacy and proposal for librarian-facilitated HL instruction and assessment occurred before the study's launch. The Institutional Review Board of WFU approved this study in July 2022 under Protocol #IRB00024753.

### Intervention

The intervention included a 60-minute workshop to assist participants in making informed decisions regarding finding, evaluating, and using online health information. A total of four workshops were available during the Fall 2022 and Spring 2023 semesters for four separate cohorts of HELPS programs; no participant attended more than one workshop during the research timeframe. All sessions were synchronous, virtual, and occurred in October 2022 and March 2023 except for one in-person session occurring in May 2023 once HELPS administrators were comfortable holding in-person sessions post-COVID.

Workshop content was facilitated by the librarian investigator and incorporated educational tools including discussions introducing HL levels as defined by Nutbeam [5] and their implications and proposed health outcomes and disparities as indicated by Squires et al. [20] in the RTI HL Conceptual Framework. For instance, instructional prompts encouraged participants to consider cultural literacy as a tangential component and acknowledge related challenges when seeking health information. Engagement activities utilizing social media platforms via break out rooms or small group discussions provided experiential learning approaches to unpacking health information messages in YouTube Short, TikTok, TED Talk, and Mayo Clinic Minute videos. Participants were exposed to lateral reading strategies, also used by expert fact-checkers, to validate video message content and presenters [29]. The lateral reading methodology involves the four “SIFT” moves (S = stop, I = investigate the source, F = find better coverage, T = trace claims to original content) where readers leave the media source and attempt to find supporting information using additional – or lateral – tabs on their device [30]. Additionally, trusted consumer health information sources were demonstrated including MedlinePlus and Drugs.com with introductions to DailyMed and ClinicalTrials.gov. The Mayo Clinic and Cleveland Clinic virtual health libraries were shared for awareness but not demonstrated. Lastly, recommendations for the MyPlate and CalorieKing mobile apps were mentioned as future additional or alternative tools to the MyFitnessPal app used by HELPS participants during the program. Rationales for choosing the selected sources were shared with participants during sessions, included in lesson plans, and derived from training, experience, and expertise of the librarian facilitator. Workshop slides, lesson plan and teaching script are available in Appendices A and B.

### Assessment and Data Analysis

Participants were invited to take a seven-question digital survey via Qualtrics before and after workshops allowing for the collection of data identifying baseline and completion HL indicators. Although various forms of validated HL measurement tools are documented in the literature, no existing tools attempting to collect attitude and behavior information from study participants were found [31]. As the objectives in this research aimed to survey the attitudes and behaviors following HL-based instruction and activities rather than to test HL levels per-post workshop, the investigator developed a new survey (Appendix C) which aimed to capture information aligning with study objectives and which addressed three themes among questions: (1) types of health information used by participants, (2) sources of health information, and (3) respondents' attitudes and behaviors prior to making health related decisions. Types of information were addressed in two categories and, therefore, separately in the survey: health and wellness (HW) and medical (MED). This delineation was determined when considering global wellness market data which compartmentalizes spending in categories. Traditional medicine and public health spending is separate from personal care & beauty, nutrition, and physical activity groups [32]. Although all categories may include overlapping health related outcomes, the author anticipated respondents may look for information on a prescribed medical test, for example, in a separate location from skin or hair care information. Sources of information and examples are provided in the survey for participants to understand and respond appropriately, i.e., WebMD is a website and PubMed is a bibliographic database. Lastly, the investigator was interested in the attitudes and behaviors of respondents prior to making health related decisions as HL can determine the efficacy associated with decision making [5,20,33].

The survey consisted of five multiple-choice / multiple-answer and Likert style questions (#1–5) aimed to collect quantitative data regarding information seeking manners as well as likelihoods and frequencies relative to trust, behaviors, and attitudes regarding health information handling. An additional open-ended question (#6) was intended to obtain various health interests and burning questions from respondents to address during or immediately after sessions. Upon data analysis and study completion, responses to this question, as well as a component of a Likert-style question addressing the frequency of engaging in trust behaviors when viewing online health information (#5c), were found interesting but beyond the scope of this research. Responses were collected, coded, and analyzed using a sequential explanatory design and various statistical measurement tools in Qualtrics, Microsoft Excel, and online calculators. Significance values were calculated for data points discussed in the results section using the Chi-squared Test which assesses correlation between variables using p-values and relies on approximation [34]. Values were considered statistically significant when p < 0.05.

## RESULTS

### Response Rates

The 65 workshop participants were invited to take part in pre- and post-surveys totaling 130 invitations and possible responses. Seventy respondents agreed to participate via informed consent and completed the survey resulting in a 53.8% response rate. Sixteen participants started but did not complete the survey and 2 denied informed consent. More participants responded to pre-workshop surveys than post: pre (n=47, 72.3%), post (n=23, 35.4%). Email invitations to participate in the survey were sent a total of four times: one week prior, one day before, immediately after, and one week after all sessions. Invitations came from the HELPS program coordinator with hopes of increasing visibility from a familiar source rather than from the librarian investigator whose name may have been unknown to participants especially in pre-session messaging. Additionally, the librarian investigator or HELPS program coordinator requested post-survey feedback during live workshops.

### Finding, Understanding, and Using Health Information

First and foremost, and in congruence with the CDCs definition of HL as “the degree to which individuals have the ability to find, understand, and use information and services to inform health-related decisions” [8], it is worth noting that 96% (n=45) of pre-workshop participants claim to find valid health information online. Ninety-six percent (n=45) of participants also claim to understand the information found and 74% (n=35) use that information to make health decisions. Post-workshop responses were not significantly different at 83% (n=19) for finding, 87% (n=20) for understanding, and 65% (n=15) for using health information online.

### Sources of Health and Medical Information

When asked where they looked for health and wellness information, pre-workshop respondents reported visiting or contacting healthcare providers most frequently (n=40, 85%), followed by mobile apps (n=34, 72%), friends/family colleagues (FFC) (n=31, 66%), websites (n=30, 64%), organizations (n=29, 62%), and social media (n=27, 57%). When asked the same question post-workshop, respondents answered similarly with a few differences: healthcare providers (n=20, 87%), organizations (n=16, 70%), websites (n=15, 65%), and mobile apps (n=12, 52%).

When looking for medical information, pre-workshop respondents were more likely to consult their healthcare providers (n=43, 91%), websites (n=32, 68%), and organizations (n=30, 64%). Post-workshop responses aligned similarly: healthcare providers (n=20, 87%), websites (n=15, 65%), and organizations (n=15, 65%).

Survey participants were guided by recommendations for each of the response options for information seeking questions. For instance, the “Healthcare providers” response included examples of such: physicians, pharmacists, therapists, physical assistants (PA), trainers.

The data indicated notable differences with responses pre- to post-workshop including a decrease in social media (-36% HW, -13% MED) and mobile app use (-20% HW, -8% MED) as well as the increase in database use (18% HW, 31% MED). The Chi-Square Test of Independence was performed to assess the correlation between these health information seeking behaviors and the workshop intervention. Pre- to post-workshop, there was a significant relationship between social media use for HW where X2(1, N=70) = 7.93, p = .005 and database use for MED where X2(1, N=70) = 9.57, p = .002. All variance and correlation values are noted in Table 1.

**Table 1:** Health information seeking behaviors pre- to postworkshop

Source / Use	Health and Wellness			Medical		
	Pre to post Variance	p-values	X^2^*	Pre to post Variance	p-values	X^2^*
Social Media	−36%	.005	7.93	−13%	.073	3.21
Database	18%	.115	2.79	31%	.350	0.87
Mobile Application	−20%	.095	2.49	−8%	.002	9.57

* For all values N=70

### Friends, Family, Colleagues, and Word-Of-Mouth Referrals

Other data worth highlighting are likelihood behaviors from respondents regarding referrals about health providers, products, or services. Referral source and behavior information was captured over seven survey response options. Only one instance of significance was found between referrals for HW information from FFC and the workshop intervention where X2(1, N=70) = 7.85, p = .005. Pre- to post- data variances are shown in Figure 4; significance values assessed using the Chi-Square Test of Independence are also noted in Table 2.

**Figure 1:**
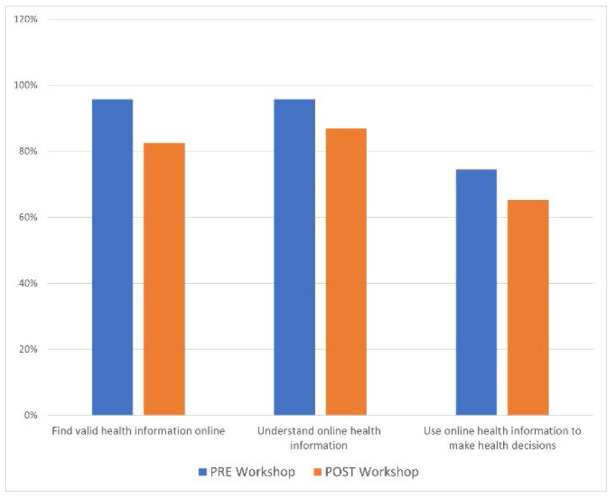
Health literacy-related information management

**Figure 2:**
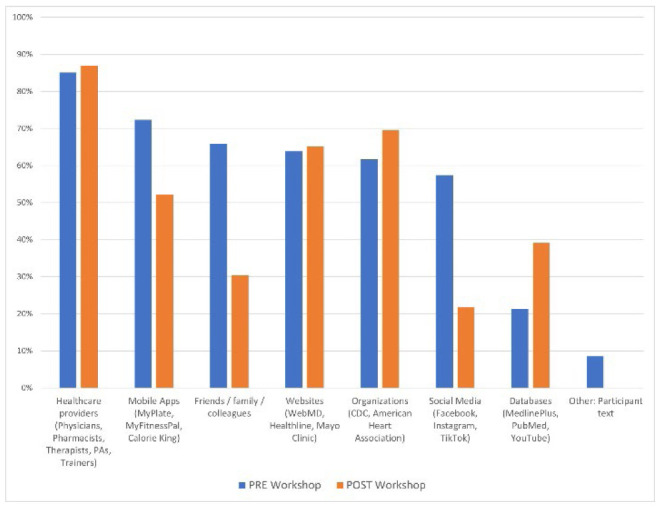
Health and wellness information seeking pre- to post-workshop

**Figure 3:**
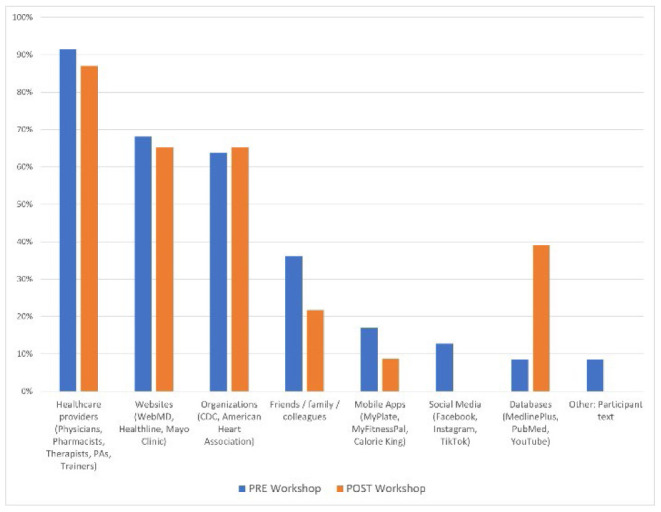
Medical information seeking pre- to post-workshop

**Figure 4:**
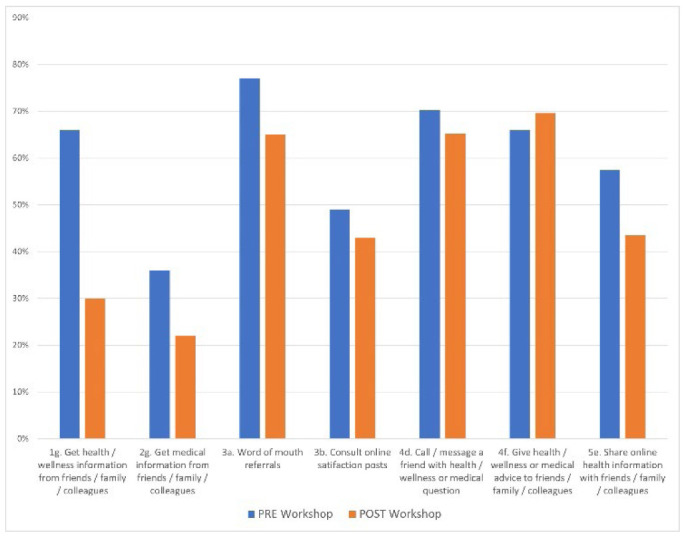
Referrals for health and wellness and medical information pre- to post-workshop

**Table 2:** Referrals source behaviors pre- to post-workshop

Survey Q#	Referral Source / Behavior	Pre to post Variance	p-values	X^2^*
1g	Get health and wellness infonnation horn friends / family / colleagues	−36%	.005	7.85
2g	Get medical infonnation from friends / family / colleagues	−14%	.222	1.49
3a	Word of mouth referrals	−12%	.315	1.01
3b	Consult online satifaction posts	−6%	.667	0.18
4d	Call / message a friend with health / wellness or medical question	−5%	.672	0.18
4f	Give health / wellness or medical advice to friends / family / colleagues	4%	.763	0.09
5e	Share online health infonnation with friends / family / colleagues	−14%	.271	1.21

* For all values N=70

## DISCUSSION

Though small-scaled, this study can invoke health science librarians and other health information professionals in pursuit of community and public health education. As instruction practices were developed using the Nutbeam [5] and RTI [20] HL models, various results aligned with some of those key concepts. Although HL was not measured in this research, attitudes and behaviors that can support and enhance HL practices were observed through pre- and post-surveys. Workshops provided a structured approach to health information seeking and response behaviors through the provision of information [33]. External factors were also addressed through practical applications offering participants exposure to lateral reading practices while combatting misinformation messages and tactics delivered through social media [20].

The employee wellness platform provided iterative instruction opportunities along with the advantage of establishing a long-term partnership between the librarian investigator and the HES department. Both entities benefitted, and continue to benefit, from the synergistic effect of a multidisciplinary partnership [21]. The HELPS program was able to expand and enhance its curriculum while the librarian provided health literacy instruction to faculty and staff who may not have been previously exposed to library services. Not only have health literacy workshops become a permanent part of HELPS sessions, the librarian liaison has since been involved in a multitude of honors and graduate student instruction and projects as well as invited to contribute to faculty research endeavors.

To address the seemingly counterproductive results reflecting declines in participants' abilities to find (-13%), understand (-9%), and evaluate (-9%) health information pre to post workshop, the author speculates variances are attributed to the Dunning-Kruger Effect where high performers tend to present inversely related confidence levels and underestimate abilities due to the awareness and acknowledgement of unknown information [35]. Although this cognitive bias-based theory is evident in multiple disciplines, Mahmood highlighted its existence when systematically reviewing information literacy skills assessment in 2016 [35]. In the case of the current research, participants may have been exposed to unfamiliar concepts and examples through workshop interventions and may have begun underestimating their confidence and abilities handling health information available through the formerly assessed platforms reviewed in the intervention.

While healthcare providers are indicated as the top source for health information among participants, websites (like WebMD, Healthline, and the Mayo Clinic) and organizations (like the CDC and the American Heart Association) are close contenders both pre- and post-workshop. Intuitively, the assumption is that responders trust these sources and are able to understand and use this information to inform health related decisions. Trust is a highly debated belief among researchers and information consumers particularly in the realm of online health information [36]. As previously noted in the Assessment and Data Analysis section, results from the Likert-style question requesting trust information from participants, are not included in results reporting of this research. These responses produced varied and insignificant results and may or may not be related to the websites, organizations, and social media responses reported in the sources for health and medical information section. Results may be due to the complex nature of trust behaviors especially when considering experiences and biases. Future research may garner some level of consistency with the existing literature. For instance, how do medical experiences or stories influence trust in online health information source selection? Or how do health information sources support and enhance trustworthy experiences for users?

More consistent and notable data was collected in this study relative to pre-post workshop differences in social media (Facebook, Instagram, TikTok), mobile app (MyFitnessPal, MyPlate, CalorieKing), and database (MedlinePlus, PubMed, YouTube) use for health information seeking. Social media and mobile app usage decreased, and database usage increased after HL workshops. The likelihood of these results could stem from both MedlinePlus demonstrations and extensive group discussions around the quality and verifiability of health messages produced on social media. Many participants admitted they were unaware of MedlinePlus as a resource for valid health information and noted its ease of use during demonstrations. Additionally, demonstrations, discussions, and lateral reading exercises provided a practical approach to vetting health information messages on social media platforms. These two primary workshop components influenced a change in a participant's approach to health information messages delivered on social media platforms. Although discussions related to mobile apps were minimal, the potential impact of quality, availability, and bias of information on free versus subscription-based apps was addressed during the workshop.

Interestingly, but perhaps not surprisingly, most respondents reported similar tendencies to obtain health provider, product, or service referrals from FFC and online satisfaction posts before and after workshops. Data suggests this age-old process in which humans participate as they gather information about anything – including health choices. Implications include the demand for more work in health literacy awareness and education at all levels being individuals, as members of families and both in-person and virtual communities, will continue to procure health information from one another.

Three major initiatives have developed from this study. First, workshops have bolstered the partnership between the library and WFU HES department. Workshop content has been embedded into HELPS employee wellness curriculums where it can continue to impact participant experiences. Although future workshops may not collect survey data before and after each session, there will now be additional opportunities for instruction method practice. Additionally, existing HELPS administered surveys can include a section relating to the HL workshop portion of the program. Second, the author has developed a curriculum for a credit-bearing library course for WFU undergraduate students: LIB290 Topics in Health Science Information. As a half-semester, 1.5-credit current course offering, LIB290 includes content driven by the HL frameworks utilized in this study; one module covers HL concepts as well as the methods utilized and findings obtained in this study. Lastly, the author is a part of the WFU Office of Civic & Community Engagement (OCCE) two-year Academic Community Engagement (ACE) Fellowship where academic instruction is strategically embedded into community programs. As a part of WFU's commitment to Winston-Salem and North Carolina, the OCCE champions community-based activity while assisting faculty and staff to practice teaching, research, or scholarship with community partners through training, development, and outreach support [37]. From 2023 – 2025, the author expects to develop and facilitate adapted HL workshops to high school students, new parents, or older adults with the intent to publish methods and findings.

## LIMITATIONS

While methods, tools, and content may offer guidance in similar academic worksite settings and wellness programs, survey responses produced statistically insignificant data (p<0.05) in most information seeking attitude and behavior indicators. Statistical significance has been reported in studies relating HL with biometric effects [16,38], the selection of weight management strategies [39,40], and micronutrient intake [14]. Although research literature document positive experiences and effects following HL interventions [12,14,16,21–25], with the complex nature of HL concepts and the acknowledgement of the many external and preexisting factors that may influence HL levels [20], statistical significance is not often an outcome of these efforts [41]. The use of convenience sampling reduced the ability to broadly generalize findings. As noted in the Methods section, workshop cohorts were predetermined and consisted of faculty and staff at a post-secondary academic institution. Several assumptions can be drawn from the nature of this sample. Accessibility, professional position and affiliation, and social and geographical demographics may contribute to survey participation and responses. For instance, a biology faculty participant who is active in their research community could be more adept to finding and scrutinizing information than a non-faculty peer in the same cohort. In this case, prior knowledge, bias, experience, and skill can pose significant limitations to data collected. The absence of a control group makes determining the impact of the intervention without considering other external factors, such as personal experiences and cultural influences, impossible. Also, a larger study population size could lend itself to more generalizable findings. Lastly, the survey created for this study has not been validated. Although the use of a new instrument was justified because no survey which captured information related to HL behaviors and attitudes was found in the literature, the lack of validation caused inherent limitations with findings which aligns with many previous attempts to quantify both HL levels and affects [42]. One example of this would be the delineation between health and wellness versus medical information appearing in two out of the six survey items. Although the author did not field questions from respondents during or after intervention sessions regarding the difference between these types of messages, they acknowledge the possibility of confusion. Repeated tests and measures could find fundamental flaws with questions requiring discernment between the two.

## CONCLUSION

Instruction and support informed by health literacy concepts are linked to improved health outcomes in employee wellness programs [12,14,16]. Specific instances of librarian-developed and facilitated curriculums in employee wellness settings, however, have yet to be documented in the literature. This study aimed to examine health information seeking attitudes and behaviors in participants before and after health literacy workshops developed and facilitated by an academic health sciences librarian. Although not without limitations particularly with sampling and the absence of a control group, intervention design and content provide examples for librarians with interests in enhancing instructional methods and building diverse partnerships. Survey results indicate the positive influence of educational and practical activities on information seeking behaviors among participants. While this study was performed in the workplace wellness environment, methods, tools, and content may be adapted to various educational settings. More practice and research are needed to further develop and disseminate validated tools and procedures related to health literacy education and in more diverse populations. These efforts can engage the public and elicit constructive health information seeking practices to inform decision making and foster favorable health outcomes.

## DECLARATION OF INTERESTS

The author reports there are no competing interests to declare.

## DATA AVAILABILITY

The data supporting the findings of this study as well as the instruments used in its execution (pre-post survey, workshop outline, sample slides) are available on the Open Science Framework at https://osf.io/dvrw8/. A project website is also available at https://sites.google.com/wfu.edu/healthinformationliteracy/home.

## AUTHOR CONTRIBUTIONS

The author confirms sole responsibility for the following: study conception and design, data collection, analysis and interpretation of results, and manuscript preparation.

## SUPPLEMENTAL FILES

**Appendix A:** Presentation Slides

**Appendix B:** Lesson Plan and Script

**Appendix C:** Survey Instrument
