# Supplementary material for: Impacts of educational interventions of librarian instruction on health information seeking attitudes and behaviors in an employee wellness program
Source: J Med Libr Assoc. 2024 May 22;112(2):107–16. doi: 10.5195/jmla.2024.1775 (PMC11305465; doi:10.5195/jmla.2024.1775)
Supplement: Supplementary file 1 — Appendix A: Presentation Slides [file jmla-112-2-107-s01.pdf]

# Health Information Literacy
# WORKSHOP

H-WFU Program Instructional Session

<Presenter>
<Presenter Title>

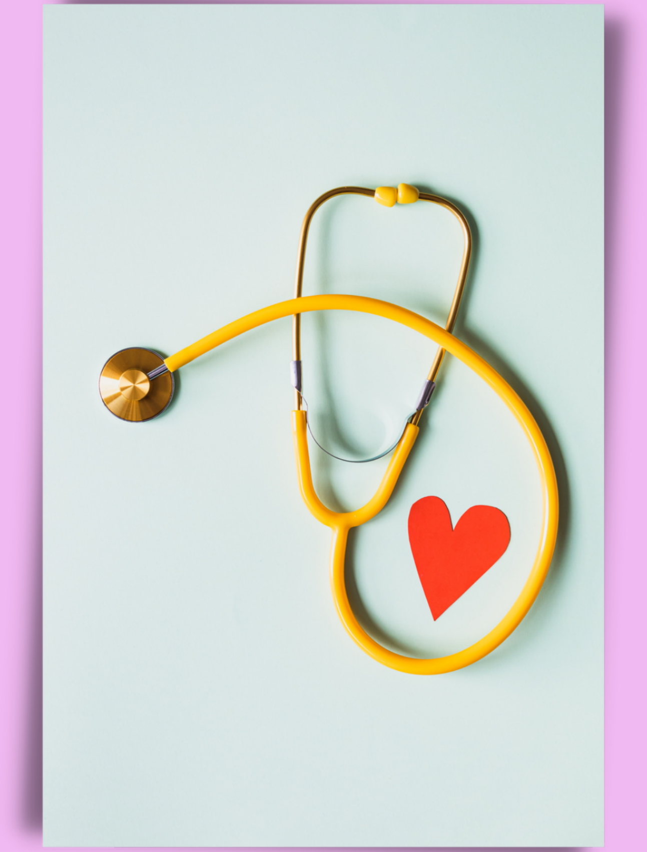

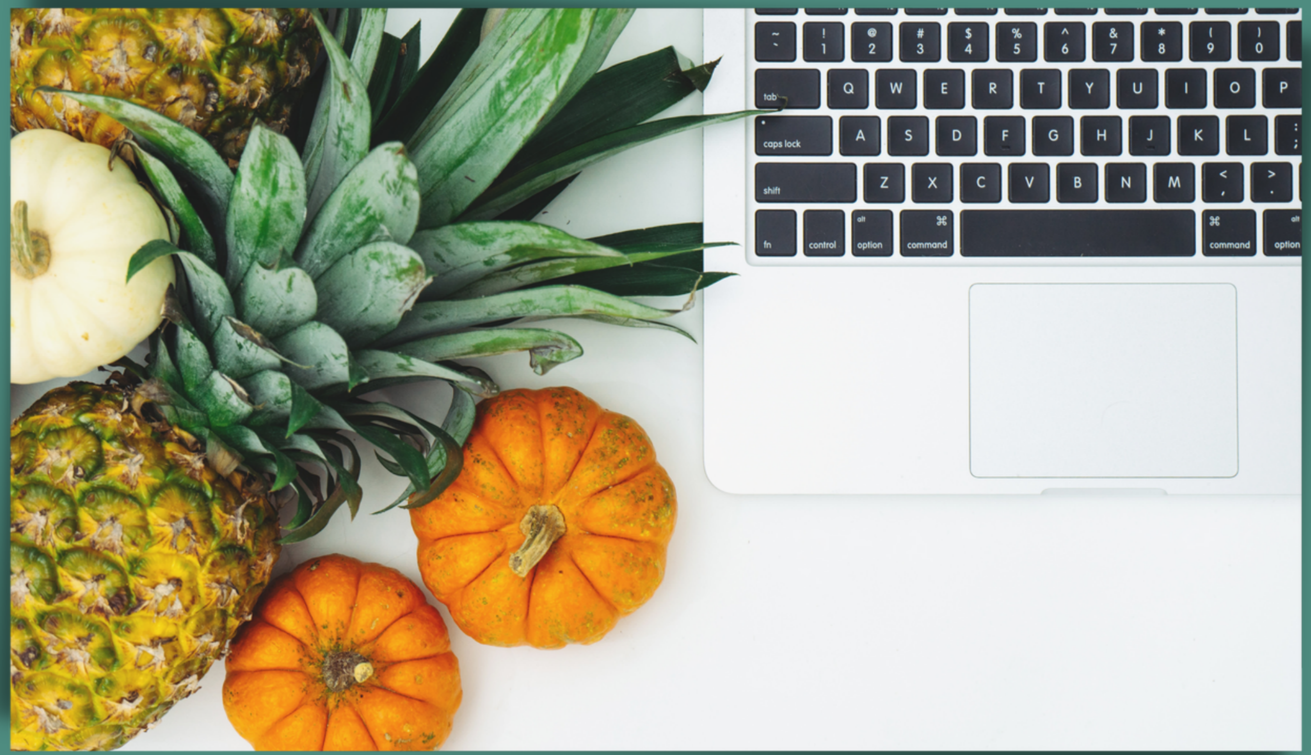

# WHAT'S AHEAD

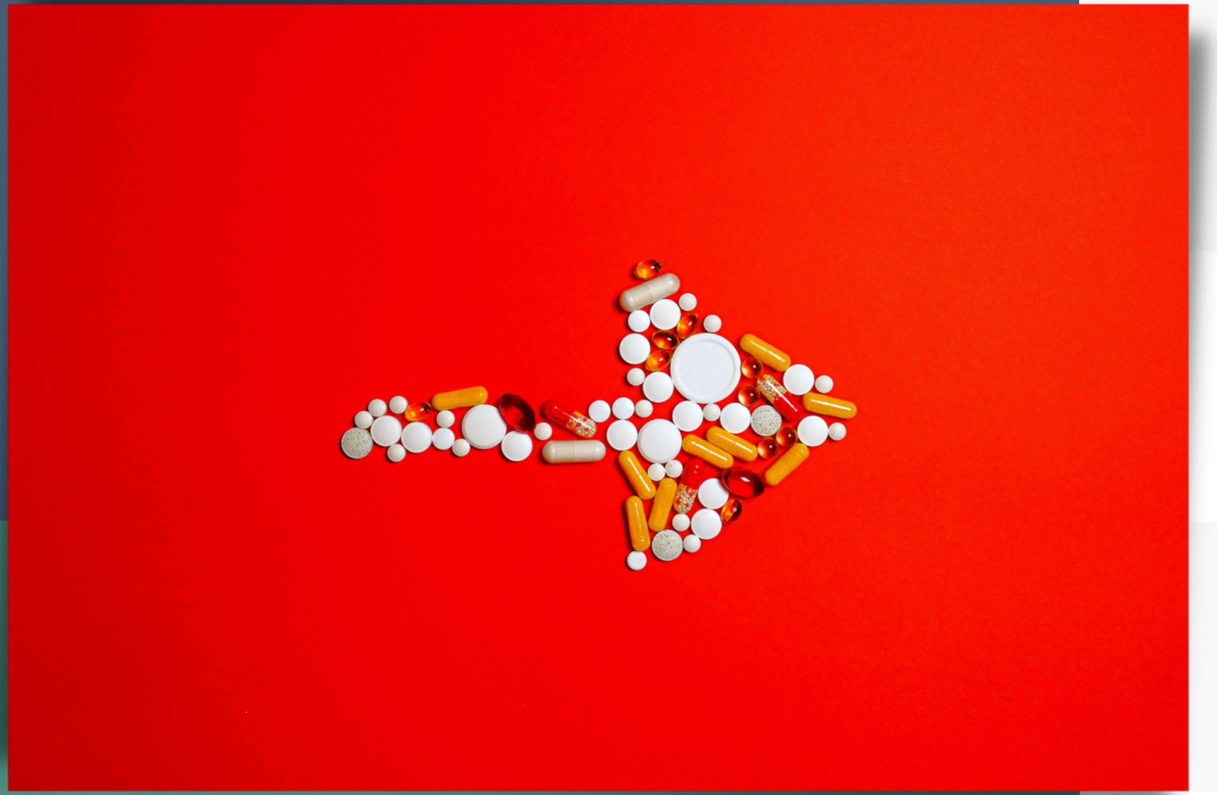

- Introduction
- What's the big deal?
- Health Messaging Activity
- Components of Health Literacy
- Health Information Sources & Demonstrations
- Recap & Questions

# INTRODUCTIONS

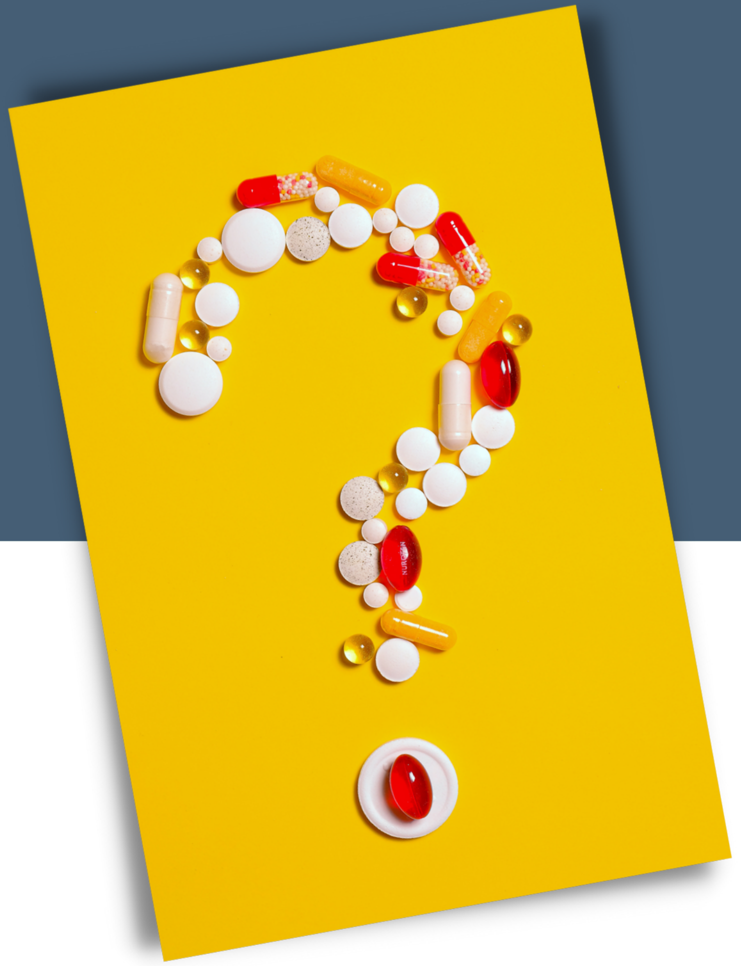

## WHO AM I?

<Redacted>

## WHY AM I HERE?

<Redacted>

# WHAT'S THE BIG DEAL?

What is Health Literacy (HL)?

## PERSONAL HL:

the degree to which **individuals** have the ability to find, understand, and use information and services to inform health-related decisions and actions for themselves and others.

## ORGANIZATIONAL HL:

the degree to which **organizations equitably enable individuals** to find, understand, and use information and services to inform health-related decisions and actions for themselves and others.

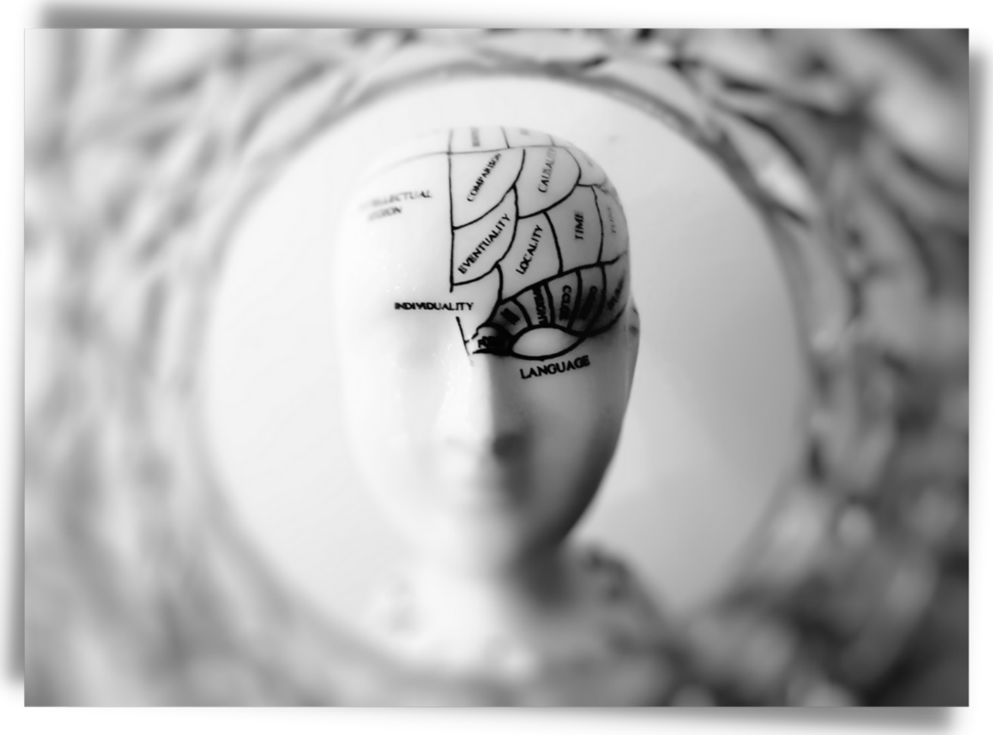

# WHAT'S THE BIG DEAL?

How does HIL impact our lives?

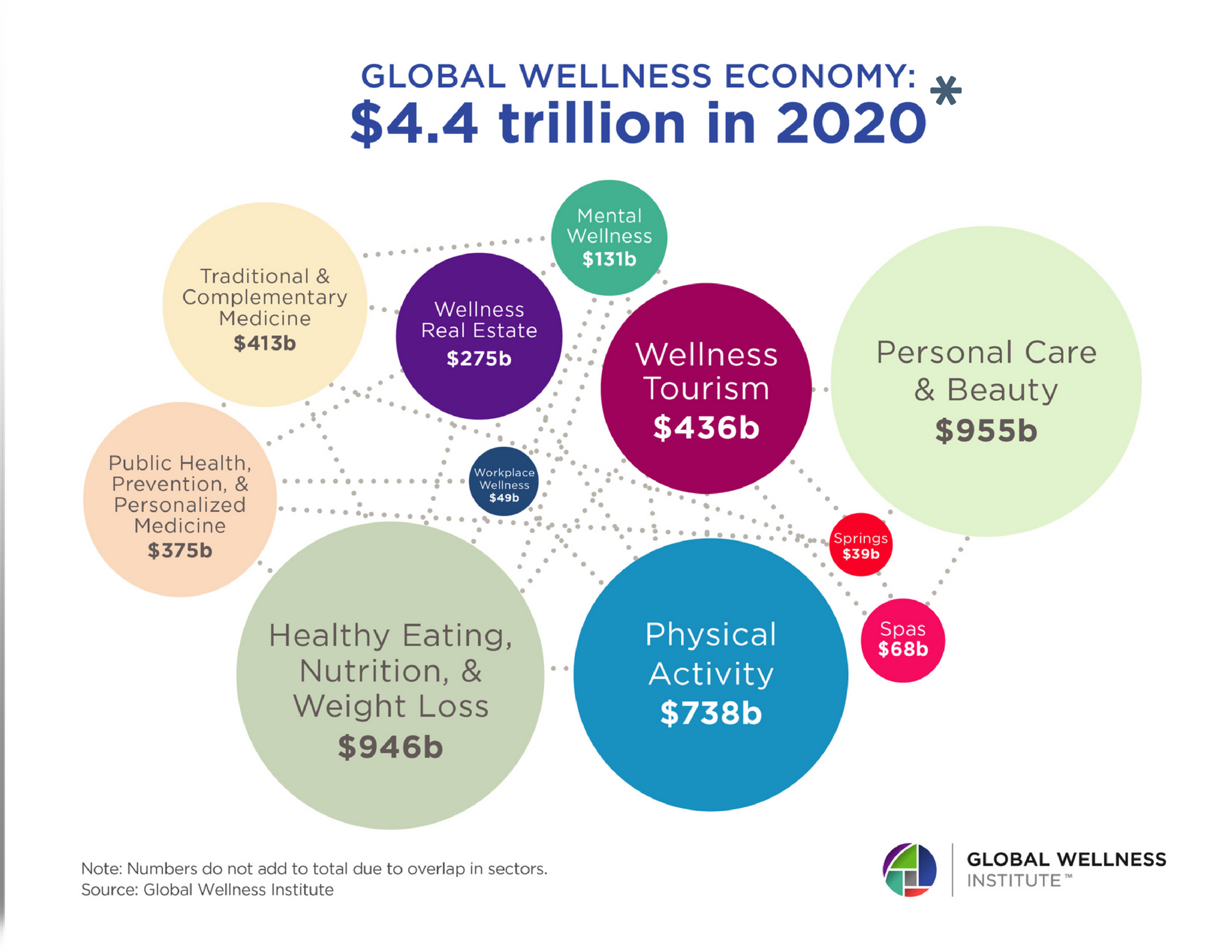


*US makes up 28% of the wellness market; China ranked 2nd at 7%!

# HEALTH MESSAGE ACTIVITY

## View

1) Choose a partner or small group

2) Watch video

## Debrief

1) Determine the validity of the message

2) Take 1 - 2 steps to determine the validity of the message

## Regroup

1) Discuss as large group

2) Share your thoughts / steps

# HEALTH MESSAGE ACTIVITY

1

ivanathealien:
Cephaly Technology to Treat Migraines

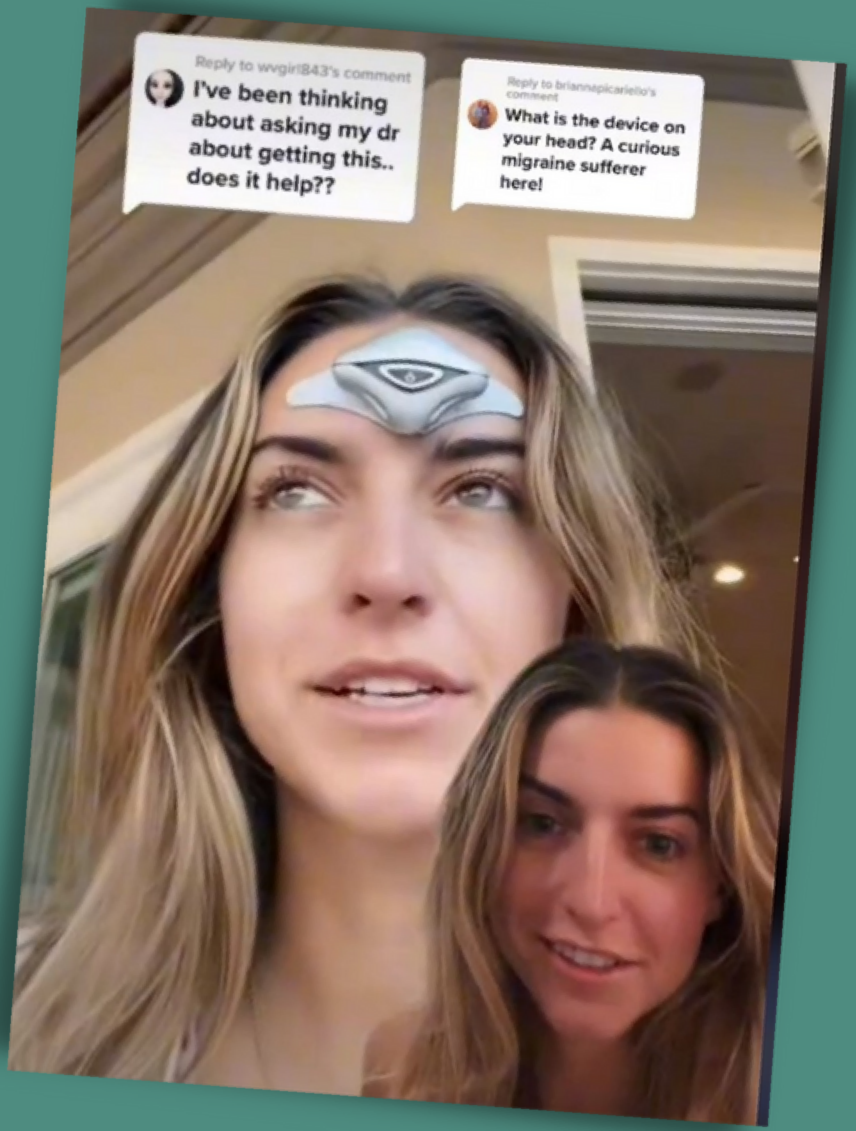


2

youngeryoudoc:
Starbucks habit unbelievably increases lifespan

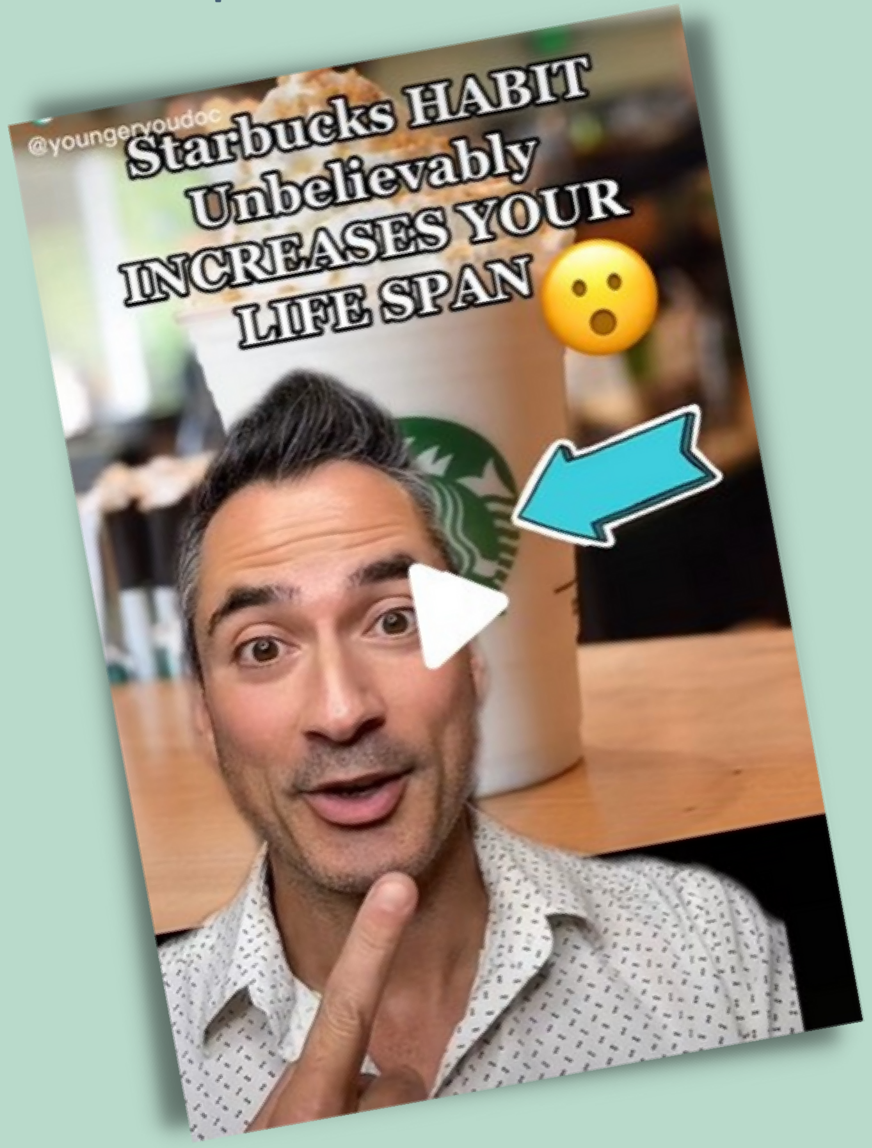

# HEALTH MESSAGE ACTIVITY

1

ivanathealien:
Cephaly Technology to Treat Migraines

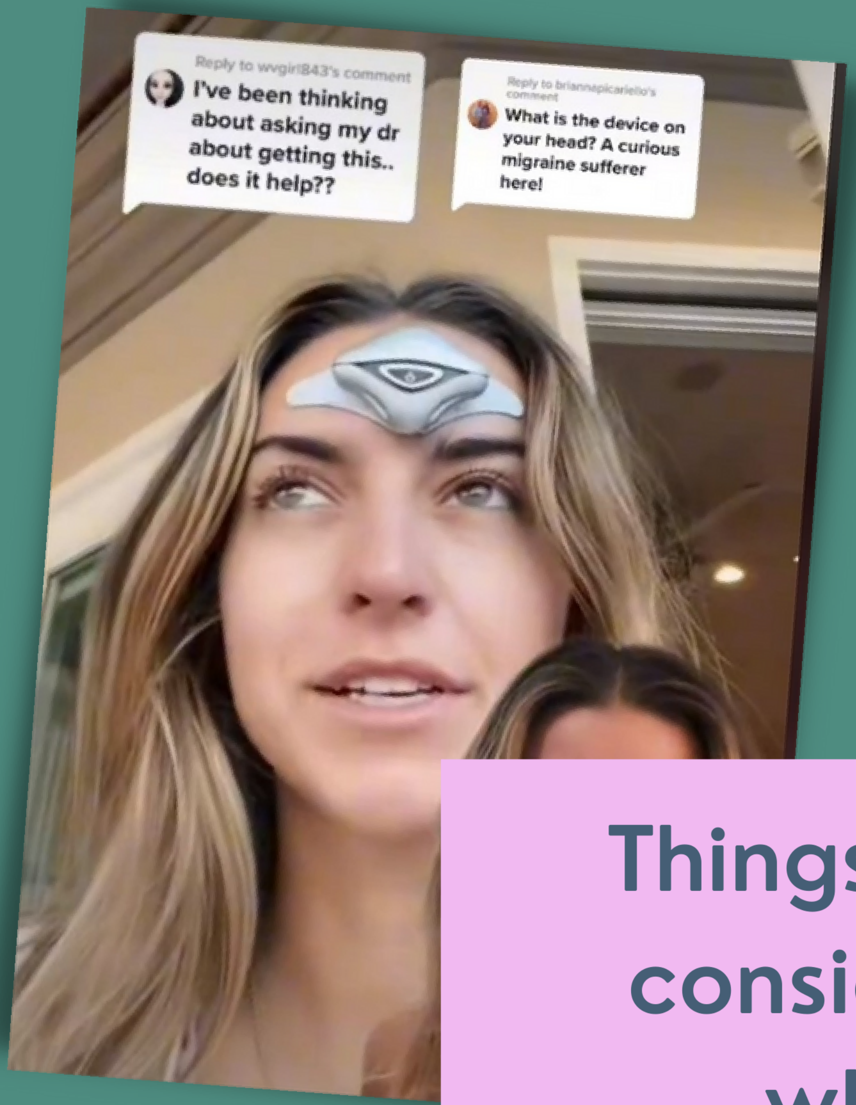


2

youngeryoudoc:
Starbucks habit unbelievably increases lifespan

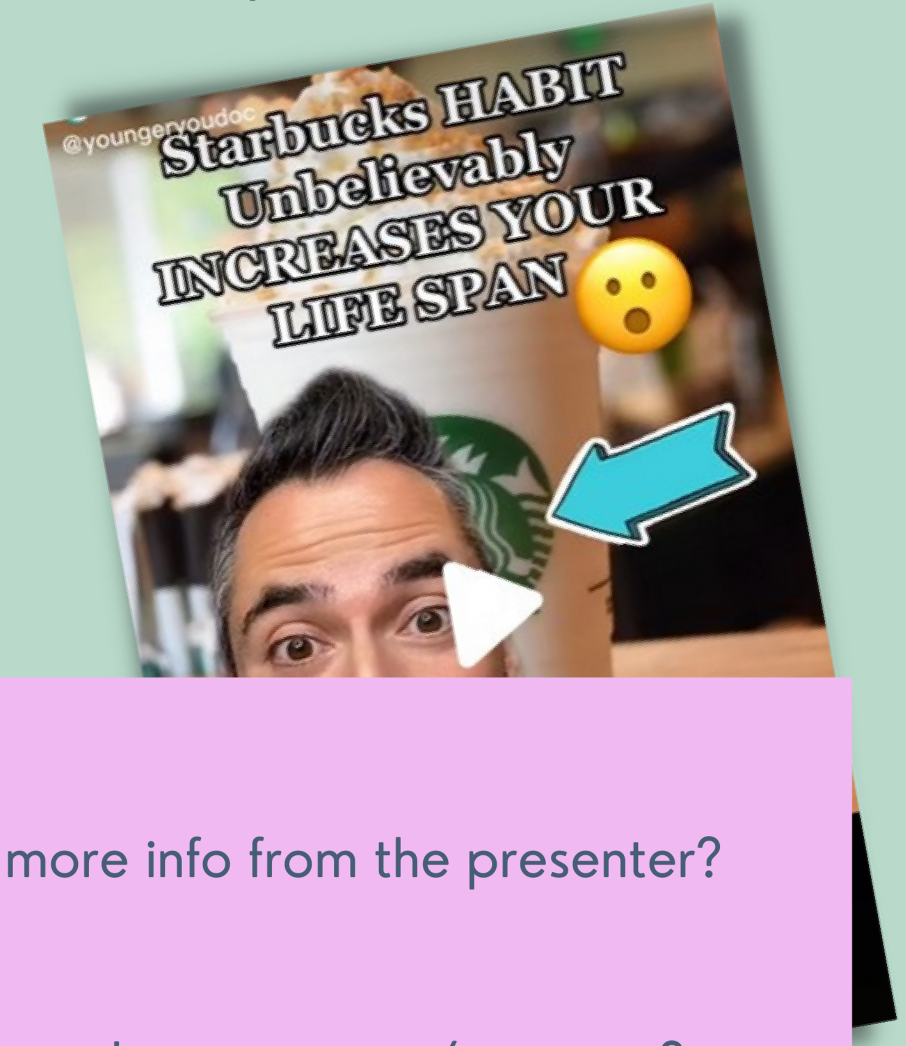


**Things to consider while watching:**

- Does this video make you want to seek more info from the presenter?
- What are the presenter's intentions?
- How long did it take to find information on the presenter / content?

# COMPONENTS OF HIL

REMINDER…

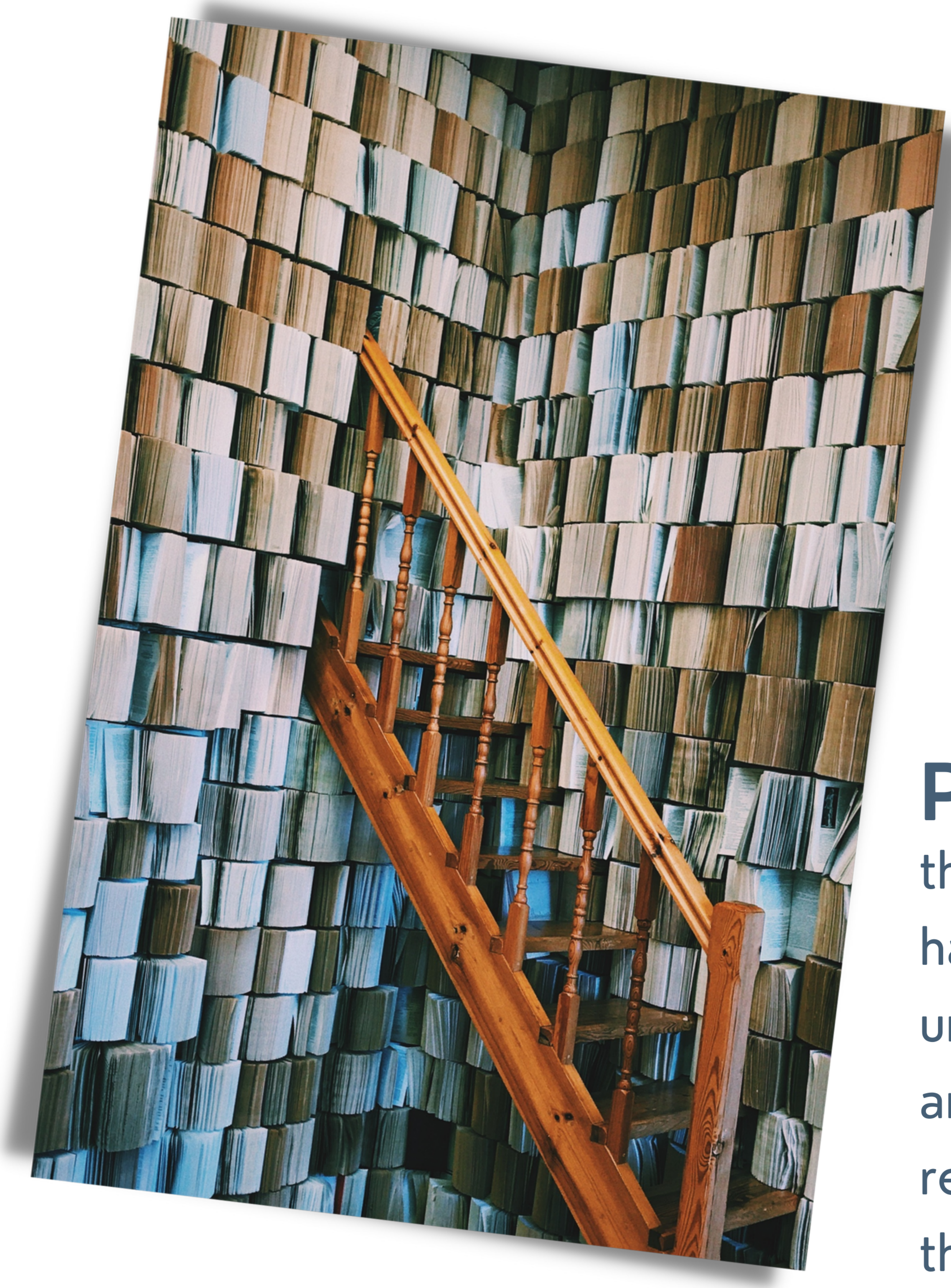

## PERSONAL HL:

the degree to which **individuals** have the ability to find, understand, and use information and services to inform health-related decisions and actions for themselves and others.

# COMPONENTS OF HIL

Level 1: FUNCTIONAL

sufficient basic skills in reading, writing, and mathematics to be able to function effectively in everyday situations.

## INCLUDES:

the ability to read and write (LITERACY), and the ability to understand and work with numbers (NUMERACY)

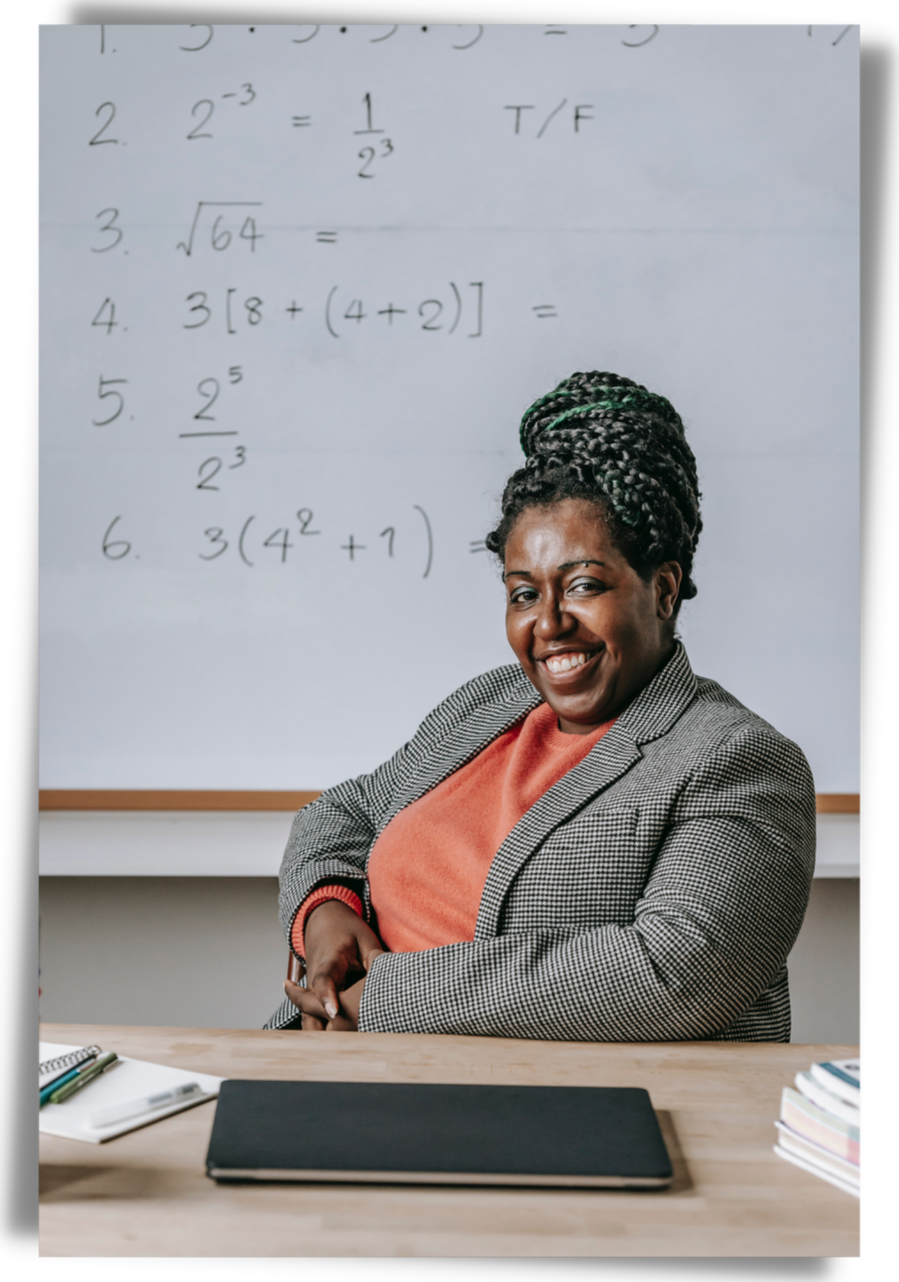

# COMPONENTS OF HIL

Level 2: INTERACTIVE

more advanced cognitive & literacy skills which, together with social skills, can be used to:

- actively participate in everyday activities,
- extract & derive meaning from communication, AND
- to apply new information to changing circumstances.

## CAN INCLUDE:

DIGITAL LITERACY: the ability to search, find, evaluate, compose clear information through typing, writing, tapping on digital platforms; requires a basic computer competencies

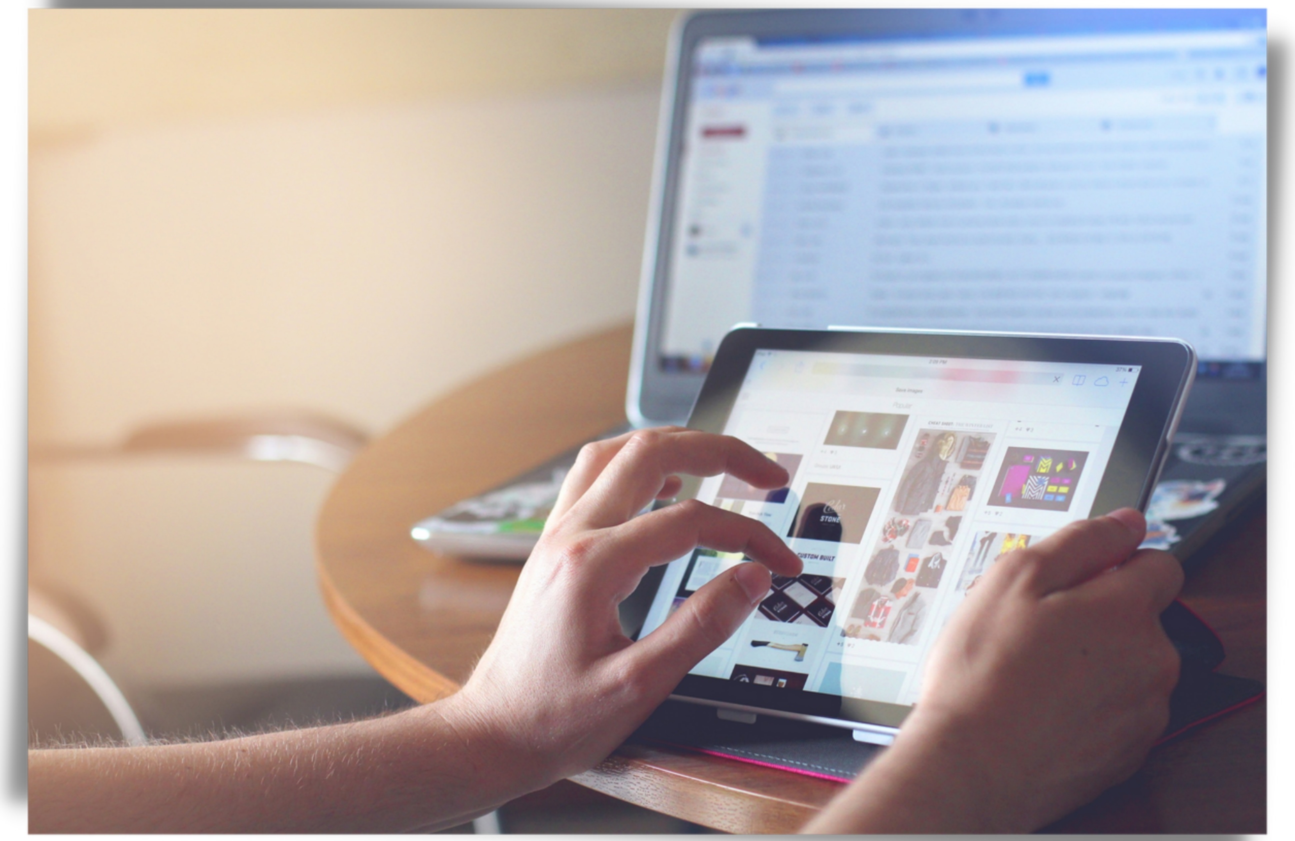

# COMPONENTS OF HIL

Level 3: CRITICAL

more advanced cognitive & social skills

- can be applied to critically analyze information
- use to exert greater control over life events

## CAN INCLUDE:

SCIENCE LITERACY: knowledge and understanding of scientific concepts and processes required for personal decision making, participation in civic and cultural affairs, and economic productivity

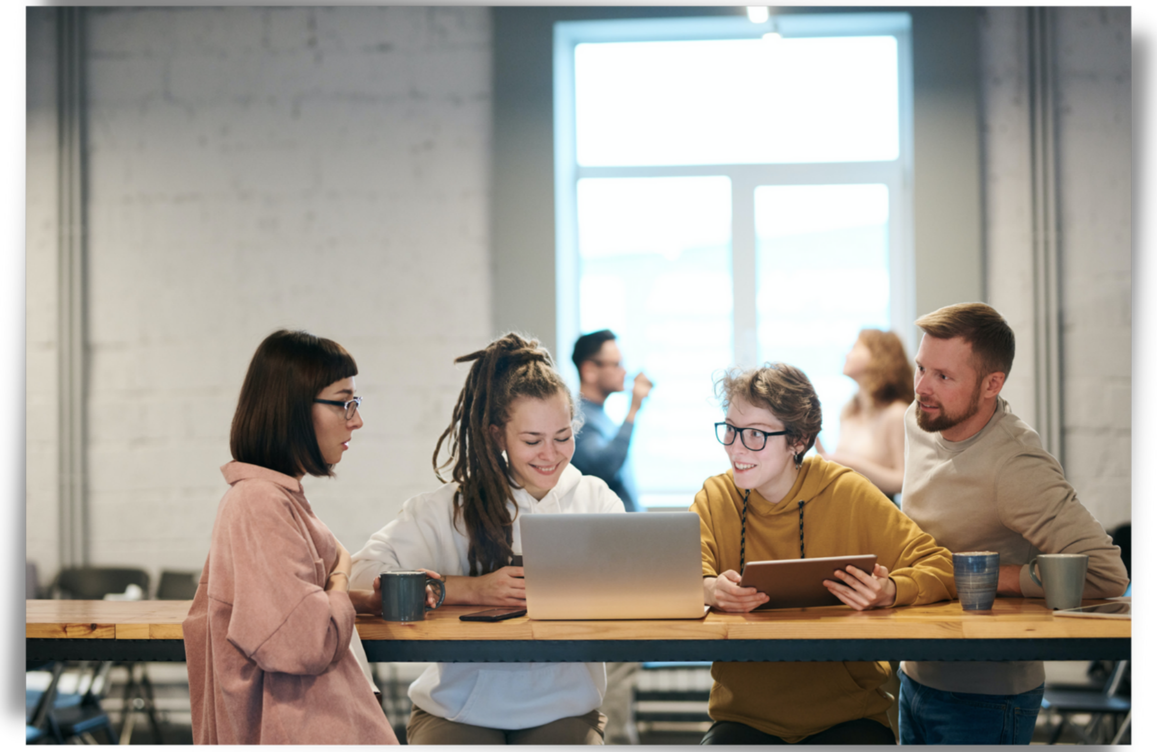

# WHAT YOU CAN DO NOW:

## INVESTIGATE

How long have they practiced?

Where have they studied? What fellowships have they been apart of?

What research have they performed?

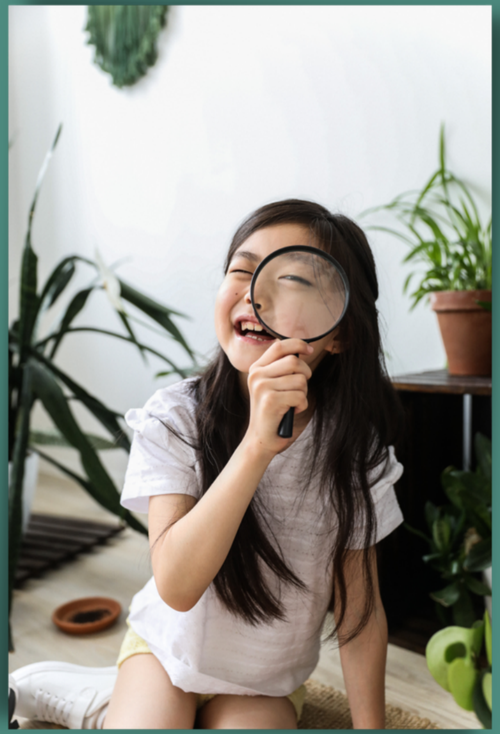

## SCRUTINIZE

Is this available in other languages (even if I don't need it)?

Could a 5th or 6th grader read this?

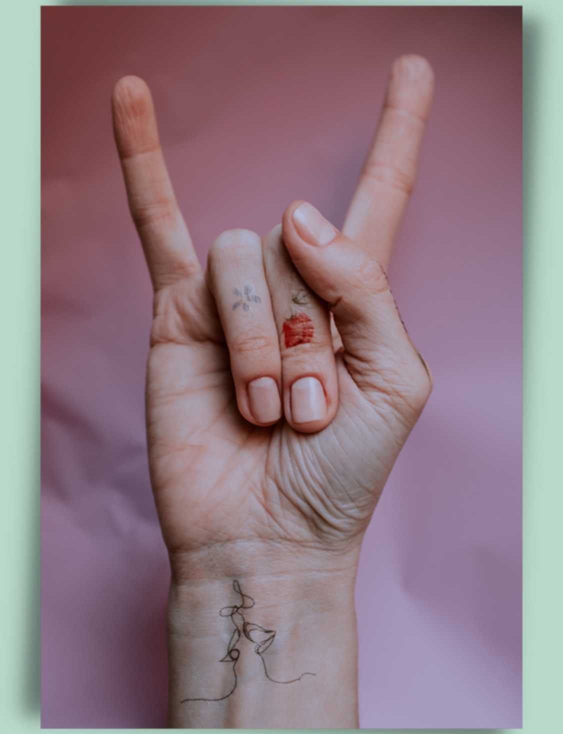

## QUESTION

Get comfortable ASKING questions

Get comfortable BEING ASKED questions

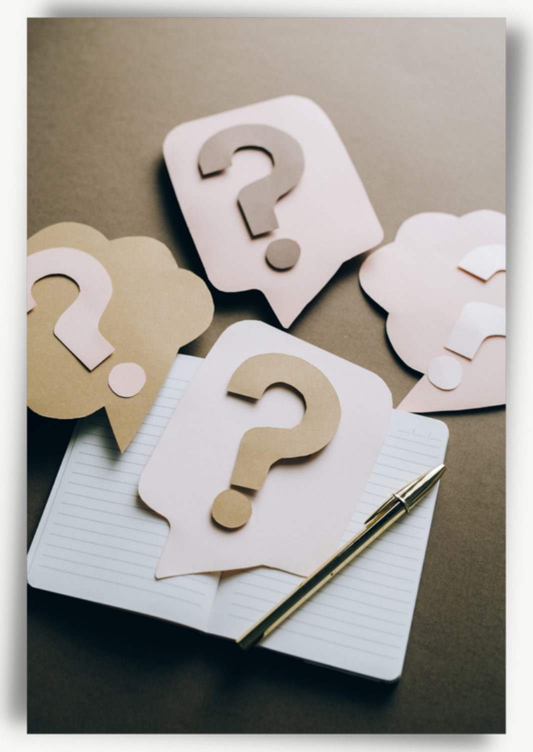

# PRE-SURVEY RESULTS

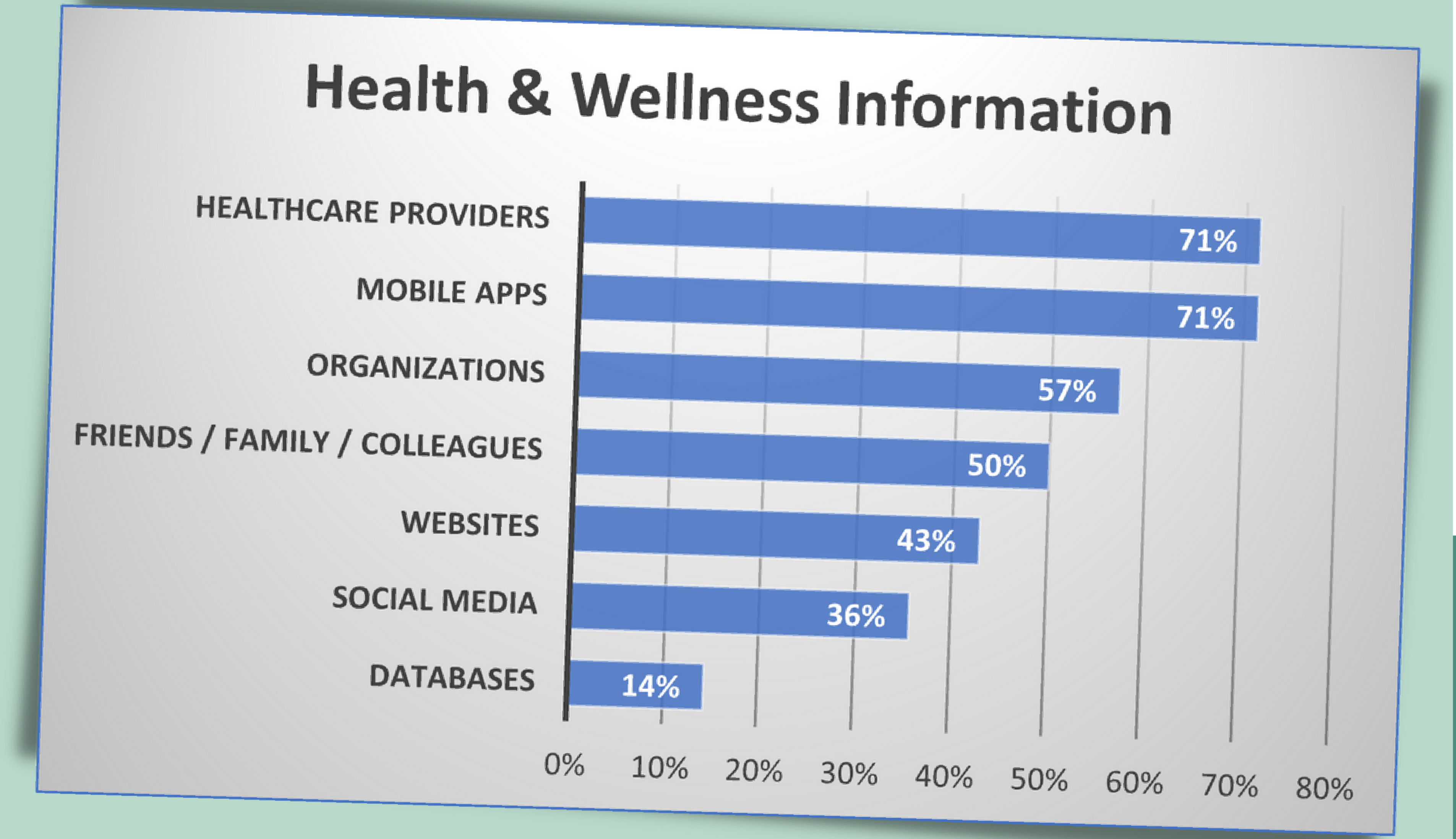


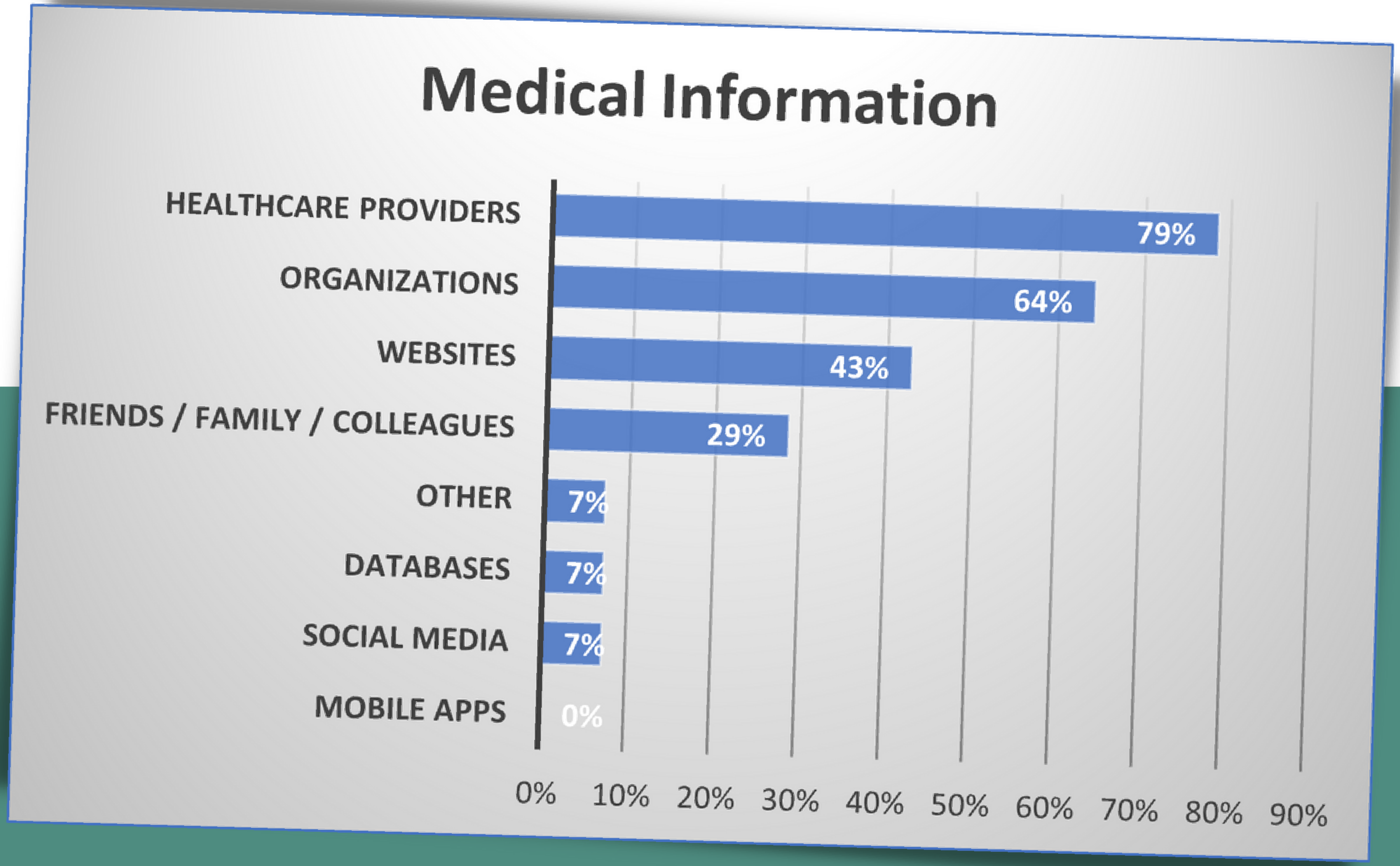

# DEMONSTRATIONS

## DATABASES

MedlinePlus®
Trusted Health Information for You

Cleveland Clinic

MAYO CLINIC

## WEBSITES

Drugs.com
Know more. Be sure.

## APPS

myfitnessspal

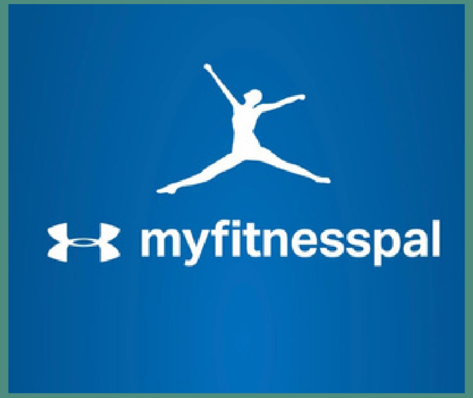


MyPlate

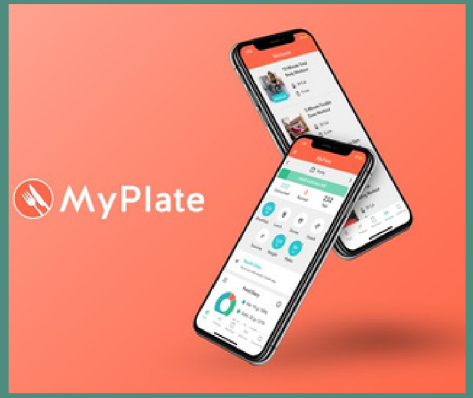


Calorie King

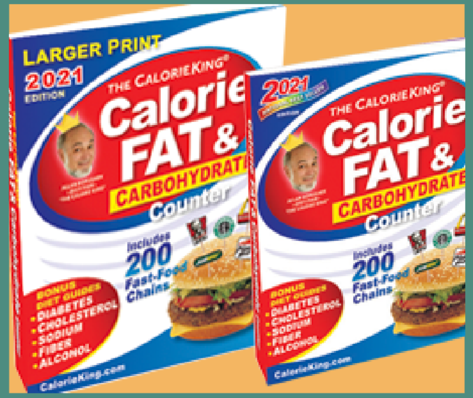

# REMINDERS:

INVESTIGATE

SCRUTINIZE

QUESTION

## HEALTH LITERACY:

"critical to empowerment"

# REMINDERS:

INV[illegible]

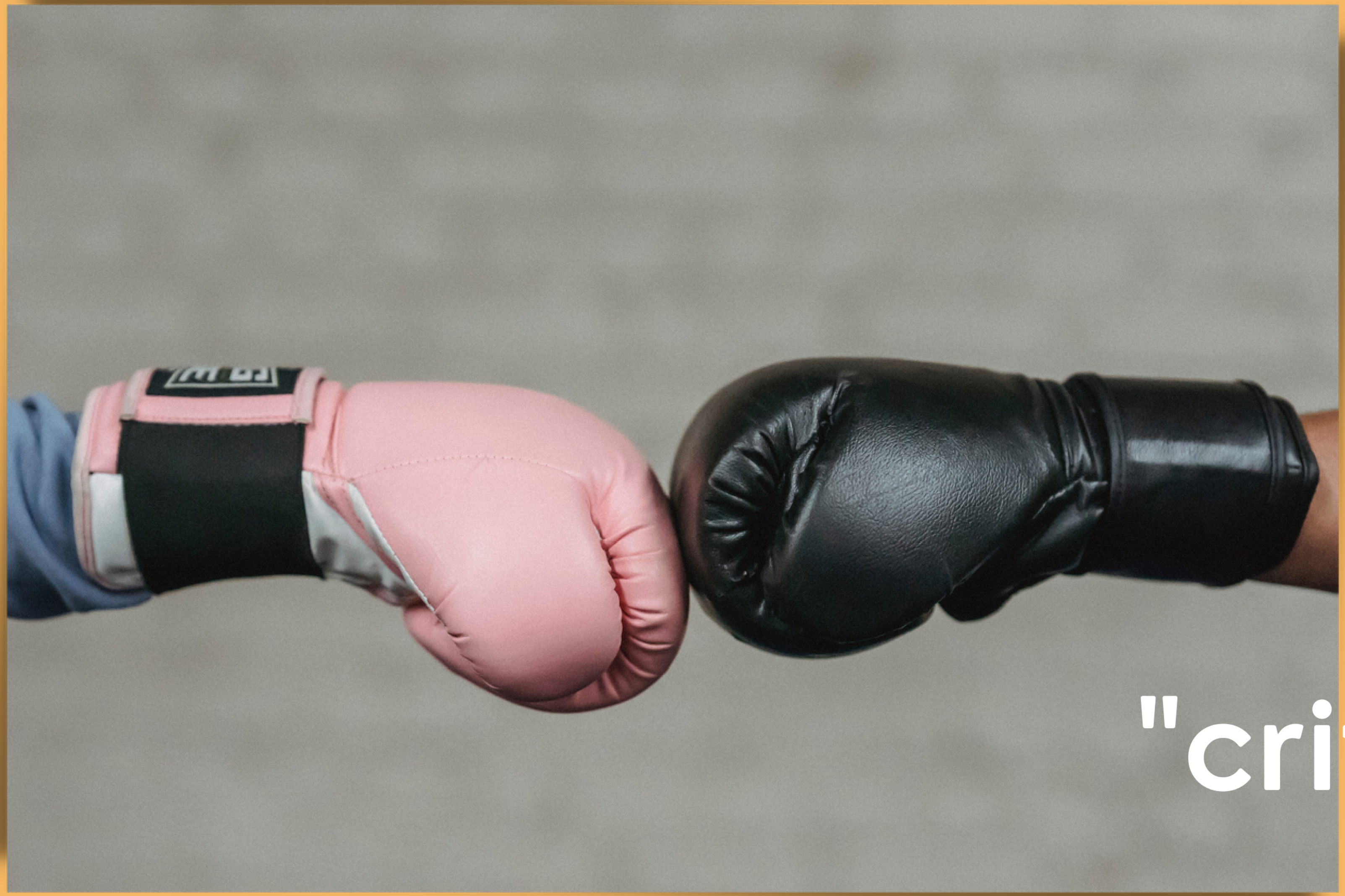

## HEALTH LITERACY:
"critical to empowerment"

## YOU GOT THIS!

# RESOURCES


Cotugna, N., Vickery, C. E., & Carpenter-Haefele, K. M. (2005). Evaluation of literacy level of patient education pages in health-related journals. *Journal of Community Health*, 30(3), 213–219. https://doi.org/10.1007/s10900-004-1959-x

Feinstein, N. (2011). Salvaging science literacy. *Science Education*, 95(1), 168–185. https://doi.org/10.1002/sce.20414

*Health Literacy*. (2015, May 8). National Institutes of Health (NIH). https://www.nih.gov/institutes-nih/nih-office-director/office-communications-public-liaison/clear-communication/health-literacy

*Mills, P. R., Kessler, R. C., Cooper, J., & Sullivan, S. (2007). Impact of a Health Promotion Program on Employee Health Risks and Work Productivity. American Journal of Health Promotion, 22(1), 45–53. https://doi.org/10.4278/0890-1171-22.1.45*

*MLA: RTI Overview. (n.d.). Retrieved September 19, 2022, from https://www.mlanet.org/p/cm/ld/fid=1333*

Nutbeam, D., McGill, B., & Premkumar, P. (2018). Improving health literacy in community populations: A review of progress. *Health Promotion International*, 33(5), 901–911. https://doi.org/10.1093/heapro/dax015

Nutbeam, D. (2000). Health literacy as a public health goal: A challenge for contemporary health education and communication strategies into the 21st century. Health Promotion International, 15(3), 259–267. https://doi.org/10.1093/heapro/15.3.259

Okan, C., Weidemann, G., & Bailey, P. E. (2021). Chapter 7 - "Alone, but not lonely": The impact of COVID-19 on older persons and the role of technology in staying connected. In A. A. Moustafa (Ed.), *Mental Health Effects of COVID-19* (pp. 117–131). Academic Press. https://doi.org/10.1016/B978-0-12-824289-6.00017-9

Raman, J., Smith, E., & Hay, P. (2013). The Clinical Obesity Maintenance Model: An Integration of Psychological Constructs including Mood, Emotional Regulation, Disordered Overeating, Habitual Cluster Behaviours, Health Literacy and Cognitive Function. Journal of Obesity, 2013, 240128. https://doi.org/10.1155/2013/240128

Scientific literacy. (2022). In Wikipedia. https://en.wikipedia.org/w/index.php?title=Scientific_literacy&oldid=1104053677

2022 *The Global Wellness Economy: Country Rankings*. (n.d.). Global Wellness Institute. Retrieved July 21, 2022, from https://globalwellnessinstitute.org/industry-research/2022-global-wellness-economy-country-rankings/

Zoellner, J., You, W., Almeida, F., Blackman, K. C. A., Harden, S., Glasgow, R. E., Linnan, L., Hill, J. L., & Estabrooks, P. A. (2016). The Influence of Health Literacy on Reach, Retention, and Success in a Worksite Weight Loss Program. *American Journal of Health Promotion*, 30(4), 279–282. https://doi.org/10.1177/0890117116639558

# WHAT ELSE?

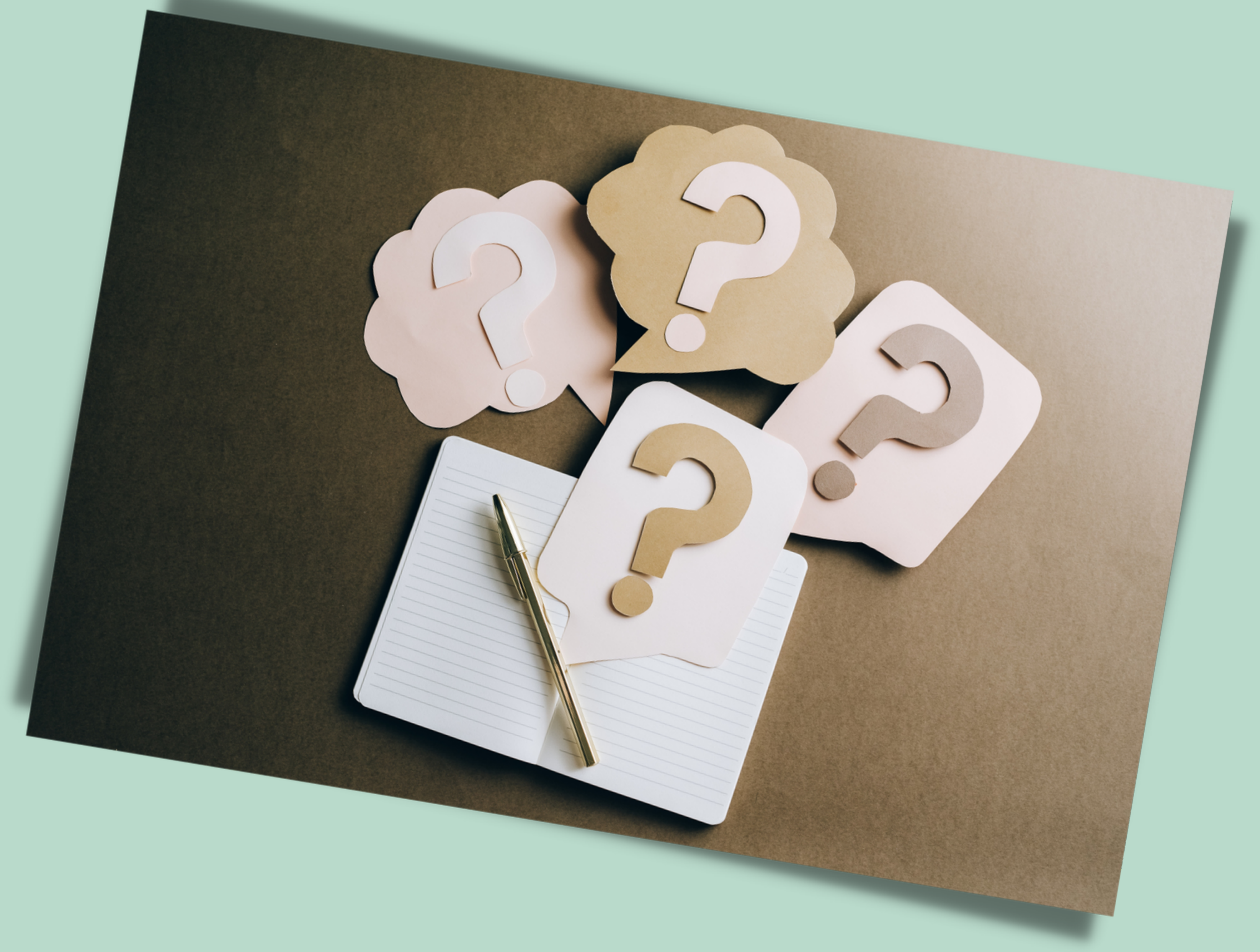

<Contact Information>
