# Supplementary material for: Impacts of educational interventions of librarian instruction on health information seeking attitudes and behaviors in an employee wellness program
Source: J Med Libr Assoc. 2024 May 22;112(2):107–16. doi: 10.5195/jmla.2024.1775 (PMC11305465; doi:10.5195/jmla.2024.1775)
Supplement: Supplementary file 2 — Appendix B: Lesson Plan and Script [file jmla-112-2-107-s02.pdf]

| HELPS: H-WFU and TLC Health Literacy Workshops |
|---|
| Outline / Talking Points / Script |
| |

I. Introduction
   A. Who am I? Why am I here?
   B. What will you expect (presentation, survey)

II. Why is this Important?
   A. What is Health Information Literacy (according to the CDC)?
   B. How does it impact our lives?

III. Health Messaging Activity
   A. Breakout groups (2 - 3) - view separate 1-minute health messages
   B. Determine validity of messages as a small group
   C. Return to full group to view messages
   D. Each group reports back to full group with findings

IV. Components of Health Literacy
   A. 3 Levels (according to Nutbeam)
   B. Components of each level
   C. Examples
   D. What can you do NOW?

V. Valid Health Information Sources Demonstrations
   A. Databases (MedLine Plus, Mayo Clinic, Cleveland Clinic)
   B. Websites (Drugs.com)
   C. Apps (MyFitnessPal, MyPlate, Calorie King)

VI. Closing
   A. Revisit purpose and intentions
   B. Request for feedback through survey participation

| | |
|---|---|
| **Slide 1 - Introduction** | 00:00 |
| Good morning! I'm <Instructor> - <Instructor Title> at <Institution> and I'll be conducting what I'm calling a HIL Workshop with you today. This is my 4th workshop over the last year so I'm determined to make this one my best one yet. | |
| **Slide 2 - Agenda** | |
| Here is today's agenda. I'll be sharing some crucial health literacy information with you including what that is of course.<br><br>We're going to do an activity to start us off and we'll wrap up with some demonstrations in databases I hope you find useful.<br><br>Please be sure to ask any questions along the way. We don't have to wait until the end for that. | |
| **Slide 3 - Who am I? And Why am I Here?** | |
| <Redacted> | |
| **Slide 4 - What's the big deal (definitions)?** | |
| This is a big deal for a number of reasons, but FIRST let's define Health Literacy (HL) just in case it's something new or you haven't heard about it in a while:<br><br>The definition of HL has evolved over the last 20 years from a former focus on individual ability - or Personal HL - to include the additional emphasis on community-centric - or organizational HL - responsibility.<br><br>So first, personal HL is the degree to which individuals have the ability to find, understand, and use information and services to inform health-related decisions and actions for themselves and others.<br><br>Organization HL is the same, however, with a responsibility on organizations to equitably enable individuals to find, understand, and use health information…<br><br>Attention to HL could avoid unnecessary hospitalizations and confusion when reading prescriptions for instance and enhance health determinants and overall wellness. And with the CDC declaring that nearly 90% of the US population lives with limited HL, | |

| | |
|---|---|
| this is really important.<br><br>We'll dive more into the components of HL later on… | |
| **Slide 5 - What's the big deal (GWM)?** | |
| Also, the Global Wellness Institute reported (preCOVID) a global wellness market of \$4.4T, with the US composing 28% of the market translating into \$1.2 trillion spent on various health and wellness aids from public and preventative health to physical activity and traditional medicine.<br><br>And this is twice as high as the second-ranked nation on the list with China spending 600b. Our culture, our access, our financial privileges make our country more apt to spend on these items. | |
| **Slide 6 - Health Message Activity** | 00:08 |
| So let's start with an activity which will help add to a list of to do's I'm hoping to help you create for when you are researching health information.<br><br>So I'd like you to partner up (or at most be in a group of 3).<br><br>In your small groups, you will:<br><br>1) VIEW a 30 - 60 second video: watch your video together - I'll share links in just a moment.<br><br>2) DEBRIEF: Within your groups, share initial thoughts / gut feelings about the message and its validity. ake 1-2 steps to confirm the validity of the message and / or the presenter<br><br>3) REGROUP: Then we'll all come back together and I'll ask you to share your group notes / thoughts / steps | |
| **Slide 7 - Groups & Video Links** | |
| So you'll be watching one of these two videos.. | |
| **Slide 8 - Things to consider** | |

<table>
<tr><td>Here are just a few things to consider as your watching and talking about your videos:<br><br>1) Does it make you want to seek more information from the presenter / platform?<br>2) What are the presenter’s intentions?<br>3) How long did it take to find information on the presenter / content?</td><td></td></tr>
<tr><td>DISCUSSION: Play Videos & discuss with each group</td><td>00:10</td></tr>
<tr><td colspan="2">Video 1: ivanathealien<br><https://www.tiktok.com/@ivanathealien/video/7060328604160363822><br><br>Who is Ivana the Alien??<br><br>What’s her MO? What are her intentions? Influencer, Spokesperson<br><br>Product? $500, maintenance costs with new electrodes. No prescription needed (good / bad)...<br>Can use FSA funds but that’s still your money<br><br>Viewer comments…<br><br>**This is risky because word of mouth referrals are a big source for health information at least with the survey feedback for this class. That’s a relatively small number, but marketing strategies timelessly rely on user feedback and referrals. This isn’t going to change in our culture.<br><br><br>Video 2: youngeryoudoc<br><https://www.tiktok.com/@youngeryoudoc/video/7148575224085089578><br><br>Who is this youngeryoudoc?? Looking at their profile we find this stopchasingweightloss.com site. If you scroll down FOREVER you find “Your Coaches" information.<br><br>Googling Jeremiah Jimerson - Chiropractor<br>So he’s merged two businesses here. Chiropractic health is under the umbrella of wellness and holistic medicines (acupuncture), so maybe it’s not too much of a stretch to link chiropracty?? And weight management.<br><br>LinkedIn - two profiles both for two additional companies or places of employment:<br>Whole Body Cryotherapy at Chill Out Charleston & Elite Performance & Pain Center</td></tr>
</table>

| | |
|---|---|
| *Coffee drinking is associated with increased longevity*, European Society of Cardiology – 1950 – publish multiple journals<br><br>Platform - TikTok… there's either a very lot or a very little that can be said about TikTok being a source for medical or wellness information.<br>● It's reaching a lot of people.<br>● Entertainment purposes only!<br>● HIGHLY exercise algorithm practices customizing your experience on the platform.<br>● TikTok determines (rather quickly) your demographic and interests.<br>● It could potentially be a great platform for anyone who wants to share safe / valid information, but we just know that's not often the case.<br><br>As a side note, if it's taking more than 1 - 2 minutes to find information about someone or something online, I'd proceed with caution. | |
| **Slide 9 Components of HL and the def of HL** | 00:30 |
| The techniques used in this exercise will help reign in the content we're exposed to. In libraries we call it LATERAL READING because you're literally opening tabs to the side of the original site you're attempting to confirm. These steps can be used for the many memes that are out there as well. Anything, you either strongly disagree OR AGREE with. Check it out first!<br><br>I want to share some supporting information, to help us add to our list of to-do's moving forward when researching health information.<br><br>Don Nutbeam, a researcher that has pioneered and published quite a bit of research on health literacy, has identified 3 successive levels of HL. An individual may have trouble reaching Level 3 before they have achieved 2 for instance. Not in every case, but in general. Understanding where you are on the hierarchy is a good start to determining your next steps and how to continue to advance your understanding & behaviors when it comes to health information analysis.<br><br>As presented earlier, and as a reminder, the definition of Personal HL is HERE...<br><br>We did talk about the organizational HL, but we're going to focus on what we can control and the next steps we can take today as consumers. | |
| **Slide 10 - LEVEL 1** | |

| | |
|---|---|
| The first level of HL is FUNCTIONAL. This is where individuals have sufficient basic skills in reading, writing, and math to be able to function in everyday situations<br><br>This includes basic LITERACY or the ability to read and write and NUMERACY, being able to work with numbers.<br><br>The recommendation for patient education materials is to be written at 5th or 6th grade levels which seems uncomplicated enough.<br><br>I coincidentally have a 5th and 6th grader at home. When I talk to them, I have an advantage though. I’ve known them for a while. I’ve seen them develop. I know the depth of topics they can handle and can anticipate what questions they may ask for the most part. This is a huge and alternate dynamic between a healthcare provider and their patients though.<br><br>Even if we've known our doctor or therapist for years, they don’t (typically) vacation with us, or have dinner or drinks every other Thursday night… there isn’t a capacity for them to get to know you on a personal level to know fully what you’re capable of understanding. AND they only have about 15 minutes with you every year (maybe more than that AND maybe less than that)?!<br><br>Spoiler alert, I’ll be throwing wrenches into each of these levels, just to exemplify how complicated the realm of HL can be for a community at large. | |
| **Slide 11 - LEVEL 2** | |
| Level 2 is INTERACTIVE or COMMUNICATIVE. This level incorporates more advanced cognitive and literacy skills which, together with social skills, can be used to actively participate in everyday tasks, to extract information and form meaning from different forms of communication, and to apply new information to changing scenarios.<br><br>This can include DIGITAL LITERACY. We're in person today, but a high percentage of our day is still spent in digital environments. Barring any inevitable technical issues that sometimes occur, we're typically able to make it to those sessions.<br><br>Other types of literacy that fall into this level are, for example, are CIVIC and CULTURAL. Did you grow up in a suburban area? Or an inner city region? Was it extremely rural? Are you from a family of immigrants? Are you a first generation American? Did you need to - for instance - at a young age how to speak English? Each | |

| | |
|---|---|
| instance poses challenges to how we view and use information now and at different times in our lives. | |
| **Slide 12 - LEVEL 3** | |
| And Lastly is LEVEL 3 - CRITICAL HL. This level requires more advanced cognitive and social skills that can be applied to critically analyze information, and to use this information to exert greater control over life situations.<br><br>About 10 years ago, University of Wisconsin - Madison professor Noah Feinstein, shared their thoughts on the benefits of science literacy in people in or pursuing non-science-based careers - calling it "public engagement with science."<br><br>There's typically no argument or It makes sense for YA when attempting to develop a certain level of financial literacy for instance. If they ever want to buy a house or start saving for retirement. But there is a lack of push for YA to develop science literacy unless they are pursuing it in higher education.<br><br>Starting in 2020, let's just face it, the world could have benefitted from having a higher HL or SL level to assist with staying healthy and understanding the ins and outs and everyday changes with the pandemic. We've seen some interesting adaptations of measures some folks were taking because they either heard or read somewhere that those were helpful practices.<br><br>So we can see, due to the many facets of HL, the many instances where information may be either misunderstood, or missed completely, when communicated. | |
| **Slide 13 - What can you do NOW?** | 00:40 |
| So we had some practice with our short videos on steps to take to be more informed.<br><br>First we can INVESTIGATE: Check credentials of spokespeople, speakers, articles, websites, etc. Particularly with websites - DON'T get information about the site from it's ABOUT page. Use Wikipedia - that's not a sin.  I teach my students how to use Wikipedia and when to use, however.<br><br>My son needed a series of arm surgeries over the past year and MY priorities were what research and fellowships his surgeon participated in. I didn't necessarily care that they looked to be just a few years older than my 12 year old. All I cared about were specific experiences they had. Someone else may want to see those years of service in | |

| | |
|---|---|
| a provider. As long as you're seeing and can validate what your definition of important is - is what matters.<br><br>Second, SCRUTINIZE!<br><br>When you receive or read health information, ask “is this available in other languages?” - even if you don't need it to be!<br><br>Can a 5th or 6th grader read it? again even if you can handle a higher level.<br><br>Advocate for your neighbors and fellow community members. Be aware for yourselves as well as for others. Community health = public health.<br><br>Lastly, QUESTION EVERYTHING!<br><br>Ask questions about things you’re not following or not understanding (for yourself or others)... this includes consulting with valid websites / databases.<br><br>If your friend or neighbor can't provide some confirmation on something they've heard, find it yourself (or at least take it with a grain of salt until you can confirm it).<br><br>Also, make sure you’re ready to answer questions - of your healthcare provider or a neighbor when you share some health information with them. Be able to back yourself up.<br><br>I realize asking questions comes with some fear or anxiety or reservations… but the more comfortable we get as a society doing so, the better informed we'll be. Any questions so far?? | |
| **Slide 14 - Intro to Databases/Websites** | 00:45 |
| So I want to share some of my top picks for valid, evidence-based health & wellness information.<br><br>Thanks to those of you who completed the pre-session survey. Here are a few notes on where this group gets their H&W and MEDICAL information. Most of you get BOTH your WELLNESS & MEDICAL information from your healthcare providers - which is great! | |

| | |
|---|---|
| The runners up are websites, organizations, AND you'll see (for medical info) friends / family / colleagues. This is similar to fall sessions when it comes to FFC. Culturally we just go to sources we trust for information regarding many things. So, for me, this information TODAY is super important so we can keep the cycle and spread of information to be valid and good information being we tend to get information from each other.<br><br>Databases are on the low end here for both (and as a librarian I highly support those). So let's get into the demos! | |
| **Slide 15 - Demonstrations** | |
| This slide shares few different trusted sites and databases for health information. I'm just going to share two of them. We'll be looking closer at MedLinePlus, but I will honorably mention the Mayo and Cleveland Clinics. They both have great patient or consumer-based libraries. They do rely on vendor donors, so you'll see ads when using those tools. The information is good however.  And I'll take just a few seconds to share Drugs.com as well.<br><br>*Switch to web for demonstrations HERE*<br><br>And lastly, as for apps, I know you all are familiar with MyFitnessPal as a part of this program. I will give an honorable mention to MyPlate. I find there are not as many ad interruptions when using MyPlate. And also, Calorie King - NOT an interactive tracker. Solely used to see Macros and calories. It is used and supported by the JDRF. I for one use it very regularly when determining insulin dosage for my 10-yr old who has Type 1. | |
| **DEMO - Script** | |
| MEDLINEPLUS - 5<br>MedLinePlus is an online information service provided by the National Library of Medicine which means librarians and other health professionals ensure the information is valid. They partner with National Institutes of Health to bring text- and video-based information and tools and is a free source!<br><br>You can start your search a number of ways. You can clink on HEALTH TOPICS and those will be listed in categories. You can start right here in the search bar. If I'm looking for information on MIGRAINES, I'll find a basic explanation, a number of related articles listed below, and specific categories of information: | |

| | |
|---|---|
| Genetics / Drugs & supplements<br><br>All sources are from NLM. you'll never see a for-profit agency sharing information even when you choose external links provided on this site.<br><br>HOME - this entire site is available in English & Spanish with the option to translate information in over 40 languages.<br><br>MedLinePlus links out to clinicaltrials.gov. You can see what studies are out there and being offered to participants who meet criteria. Use example: MIGRAINE.<br><br>Also is this video and tools link to videos that are typically less than 2 minutes for a good overview rather than reading.<br><br>I'll urge you to try MedlinePlus when you want to understand a disorder, drug, or test procedure. You won't have to guess or take additional steps to fact check this source.<br><br>DRUGS.COM - 2<br>Any .com may get a bad wrap, but I like this site for medication information.<br>They do use ads, so ignore those. They also however have a RSS feed from the FDA which is helpful.<br><br>The pill identifier is very useful: AM, white, oval…<br><br>Interactions checker: Statin and ampyra<br>The phonetic search is good too: ALPRA You can hit enter and see a quick description to narrow down your search.<br>+Veterinary drugs (if pets are a part of your family) Carprofeb<br><br>APPS -<br>MyFitnessPal (used in HELPS programming)<br>MyPlate - minimal ads<br>Calorie King - NOT an interactive tracker. Solely used to see Macronutrients and calories… used and supported by the JDRF | |
| **Slide 16 - Reminders** | 00:55 |
| So as we wrap up here, please remember these first 3 steps you can start taking now: INVESTIGATE, SCRUTINIZE, QUESTION information you see and hear. | |

| | |
|---|---|
| As a side note, there's a lot more to this topic than we covered today. For instance we didn't talk in depth about variables relative to background and biases. Trust is a big issue in various / many communities. Historical (and current) events that plague underrepresented or marginalized groups and effect how they receive and analyze information.<br><br>But nonetheless, HL is noted as - one researcher stated it being - "critical to empowerment" when it comes to wellbeing and health related decision making. | |
| **Slide 17 - You got this!** | |
| You all are certainly taking empowering steps just by being a part of HELPS programs and acknowledging there is always more learning and work to be done when it comes to your health and longevity.<br><br>So I'll leave you with a quick YOU GOT THIS moving forward. | |
| **Slide 18 - Resources** | |
| I have plenty of resources to share - these just support what I shared today and are available if anyone is interested. | |
| **Slide 19 - Contact** | |
| Please reach out to me if you'd like to chat. I'm always happy to talk about research that's out there and any potential research on this topic.<br><br>Thank you for having me today. I appreciate your attention and participation.<br><br>And as a reminder, you'll get a second invitation to complete my survey in a few weeks. It is designed to be taken twice - once before and once after today's session. If you missed out on taking it prior to today, you can still take it when you see the link again in a few weeks.<br><br>ANY QUESTIONS?? | |
