# Supplementary material for: Impacts of educational interventions of librarian instruction on health information seeking attitudes and behaviors in an employee wellness program
Source: J Med Libr Assoc. 2024 May 22;112(2):107–16. doi: 10.5195/jmla.2024.1775 (PMC11305465; doi:10.5195/jmla.2024.1775)
Supplement: Supplementary file 3 — Appendix C: Survey Instrument [file jmla-112-2-107-s03.pdf]

Health Literacy Workshops in Employee Wellness Programs: Pre / Post Survey

1. Multiple Choice: Where do you get your health & wellness information? Choose all that apply (examples of each are included):
    a. Websites (WebMD, Healthline, Mayo Clinic)
    b. Social Media (Facebook, Instagram, TikTok)
    c. Healthcare providers (Physicians, Pharmacists, Therapists, PAs, Trainers)
    d. Organizations (CDC, American Heart Association)
    e. Mobile Apps (MyPlate, MyFitnessPal, Calorie King)
    f. Databases (MedlinePlus, PubMed, YouTube)
    g. Friends / family / colleagues
    h. Other: ____________

2. Multiple Choice: Where do you get your medical information? Choose all that apply (examples of each are included):
    a. Websites (WebMD, Healthline, Mayo Clinic)
    b. Social Media (Facebook, Instagram, TikTok)
    c. Healthcare providers (Physicians, Pharmacists, Therapists, PAs, Trainers)
    d. Organizations (CDC, American Heart Association)
    e. Mobile Apps (MyPlate, MyFitnessPal, Calorie King)
    f. Databases (MedlinePlus, PubMed, YouTube)
    g. Friends / family / colleagues
    h. Other: ____________

3. Multiple Choice: What information do you attempt to find before choosing a physician or health / wellness service or product? Choose all that apply:
    a. Word of mouth / friend referral
    b. Online satisfaction posts
    c. Education (Training) and / or publication information about the professional / product / service
    d. Amazon / Vendor customer comments / suggestions
    e. Insurance coverage
    f. Cost
    g. Other: ____________

4. Likert Scale: How likely are you to do the following? Choose one option: Extremely likely, Somewhat likely, Neither likely or unlikely, Somewhat not likely, Not at all likely
    a. Click on a pop up link to a weight loss or wellness program / suggestion when online?
    b. Purchase a weight loss program or wellness product online?
    c. Call / message a healthcare professional with a health or medical question?
    d. Call / message a friend with a health or medical question?
    e. Call / message your insurance company with a coverage question?
    f. Give health or medical advice to family / friends / colleagues / others?
    g. Share your health successes using social media?

   h. Share your health struggles using social media?

5. Likert Scale: How often do you engage in the following experiences: Choose one option: Extremely likely, Somewhat likely, Not sure, Somewhat Unlikely, Extremely unlikely
   a. Finding valid health information online
   b. Understanding health information found online
   c. Trust online health information
   d. Use online health information to make health decisions
   e. Share online health information with family / friends / colleagues / others

6. Short answer: What health topic(s) do you want to learn more about? Include up to 5 topics.
